# Supplementary material for: Single postoperative infusion of zoledronic acid to improve patient-reported outcome after hip or knee replacement: study protocol for a randomised, controlled, double-blinded clinical trial
Source: BMJ Open. 2020 Sep 30;10(9):e040985. doi: 10.1136/bmjopen-2020-040985 (PMC7528432; doi:10.1136/bmjopen-2020-040985)
Supplement: Supplementary data [file bmjopen-2020-040985supp001.pdf]

Patientens namn: ……………………..

Studienummer: ……………………..

Patientinformation

Du har artros i en höft- eller knäled. Tillsammans med din behandlande läkare har ni kommit fram till att du behöver opereras med en protes.
Denna patientinformation är en förfrågan om att delta i en forskningsstudie.

Zoledronat är ett läkemedel registrerat för behandling av benskörhet. Det verkar genom att minska den naturliga nedbrytningen av skelettet som sker med åldern. Läkemedlet verkar också kunna förbättra fastläkning av ledproteser.

Syftet med denna studie är att ta reda på om Zoledronat kan förbättra resultat efter protesoperationer i höft- eller knäled. Vi planerar att inkludera 1000 patienter som behöver en höft- eller knäprotes på grund artros. Hälften av patienterna får behandling med Zoledronat dagen efter operation och den andra hälften får endast koksalt (overksamt läkemedel). Behandlingen sker genom en långsam injektion (infusion). I övrigt påverkas inte ditt omhändertagande före eller efter operationen, utöver att vi kommer att kontrollera den opererade leden med röntgen efter 3 år och 6 år. Du kommer också att få 2 formulär hemskickat till dig vid 1, 3 och 6 år efter operation. Med dessa formulär vill vi på ett standardiserat sätt få reda på hur du upplever resultatet av din operation.

Behandling med Zoledronat kan ge biverkningar. Vanliga biverkningar efter den första infusionen är feber och huvudvärk (influensaliknande). De flesta av dessa biverkningar uppträder inom de tre första dagarna efter behandlingen och upphör inom 3 dagar efter att de började. Genom den smärtlindring du får på grund av operationen kommer du sannolikt inte märka dessa biverkningar alls. Sällsynta och allvarliga biverkningar vid behandling med Zoledronat är frakturer i lårbenet och nedbrytning av käkbenet. Dessa förekommer dock endast vid upprepade behandlingar och i kombination med allvarliga grundsjukdomar. I denna studie ges zoledronat som en engångsdos, dagen efter din operation.

Om du vill delta, kommer du att lottas till antingen behandling med placebo (koksalt) eller Zoledronat. Varken Du eller din opererande läkare vet vilken behandling Du får.

Ditt deltagande är helt frivilligt. Om du väljer att inte delta kommer du att omhändertas på sedvanligt sätt. Du kan när som helst under studien avbryta ditt deltagande utan att det påverkar din behandling.

Version 1.5 2019-04-01

Om Du beslutar dig för att avbryta studien, kommer din doktor att be dig om tillåtelse att samla in information från dina journalhandlingar. Studien är godkänd av Etikprövningsnämnd och tillstånd har lämnats av Läkemedelsverket. Du är som patient försäkrad genom Läkemedelsförsäkringen och Patientskadelagen.

Behandling av personuppgifter

Under studien kommer ansvarig läkare att samla in uppgifter om födelsedatum, kön, hälsodata (såsom t.ex. tidigare sjukdomar och läkemedelsanvändning) samt resultat av undersökningar i studien.

Uppgifter insamlas i studien utan ditt namn eller personnummer men med en kod. Endast ansvarig läkare har tillgång till din ”nyckel”, med vilken det går att koppla uppgifterna till dig. Capio Specialistvård i Motala AB är personuppgifts-ansvariga för behandling av personuppgifter.

Ändamålen med detta register är forskning och utveckling av läkemedel som beskrivits i denna information samt godkännande/registrering av kommande produkter och säkerhetsuppföljning, därmed är allmänt intresse den rättsliga grunden för hantering av personuppgifter. Resultat kan också komma att publiceras i någon medicinsk tidskrift utan att din identitet uppges.

Uppgifterna hanteras enligt Dataskyddsförordningen, GDPR (EU 2016/679) och du har rätt att få veta vilka uppgifter som samlas in om dig, begära rättelse vid eventuella felaktigheter eller begära begränsning/ borttagning av uppgifter.

Ansvarig läkare:
Håkan Ledin, Mobil: 072-204 56 35; hakan.ledin@regionostergotland.se
Bengt Horn, Mobil: 070-158 63 54 Bengt.Horn.af.Aminne@regionostergotland.se

Skriftligt Samtycke

Jag har tagit del av informationen och accepterar att deltaga i studien. Jag har också informerats om och samtyckt till att en oberoende granskare (monitor) och läkemedelsmyndighet vid behov får jämföra de i studien rapporterade uppgifterna med de som finns i min patientjournal. Detta får ske under förbehåll att den information som då blir tillgänglig inte förs vidare.

Underskrift Patient Namnförtydligande Datum

Underskrift Läkare Namnförtydligande Datum

Version 1.5 2019-04-01
