# Supplementary material for: Single postoperative infusion of zoledronic acid to improve patient-reported outcome after hip or knee replacement: study protocol for a randomised, controlled, double-blinded clinical trial
Source: BMJ Open. 2020 Sep 30;10(9):e040985. doi: 10.1136/bmjopen-2020-040985 (PMC7528432; doi:10.1136/bmjopen-2020-040985)
Supplement: Author's manuscript [file bmjopen-2020-040985.draft_revisions.pdf]

BMJ Open

BMJ Open is committed to open peer review. As part of this commitment we make the peer review history of every article we publish publicly available.

When an article is published we post the peer reviewers' comments and the authors' responses online. We also post the versions of the paper that were used during peer review. These are the versions that the peer review comments apply to.

The versions of the paper that follow are the versions that were submitted during the peer review process. They are not the versions of record or the final published versions. They should not be cited or distributed as the published version of this manuscript.

BMJ Open is an open access journal and the full, final, typeset and author-corrected version of record of the manuscript is available on our site with no access controls, subscription charges or pay-per-view fees (http://bmjopen.bmj.com).

If you have any questions on BMJ Open's open peer review process please email info.bmjopen@bmj.com

BMJ Open

A single postoperative infusion of zoledronic acid to improve patient reported function after hip or knee replacement: study protocol for a randomized, controlled, double-blinded clinical trial.

Journal:	*BMJ Open*
Manuscript ID	bmjopen-2020-040985
Article Type:	Protocol
Date Submitted by the Author:	28-May-2020
Complete List of Authors:	Brandt, Jonathan; Capio Specialistvård Motala, Department of Orthopaedic Surgery<br>Ledin, Håkan; Capio Specialistvård Motala, Department of Orthopaedic Surgery<br>Ranstam, Jonas; Lunds Universitet, Clinical sciences<br>Roos, Ewa; Syddansk Universitet Det Sundhedsvidenskabelige Fakultet, Sports Science and Clinical Biomechanics<br>Aspenberg, Per; Linköping University Hospital, Department of Orthopaedics in Linköping, and Department of Biomedical and Clinical Sciences, Linköping University<br>Schilcher, Jörg; Linköping University Hospital, Department of Orthopaedics in Linköping, and Department of Biomedical and Clinical Sciences, Linköping University
Keywords:	Adult orthopaedics < ORTHOPAEDIC & TRAUMA SURGERY, Hip < ORTHOPAEDIC & TRAUMA SURGERY, Knee < ORTHOPAEDIC & TRAUMA SURGERY

SCHOLARONE™
Manuscripts

I, the Submitting Author has the right to grant and does grant on behalf of all authors of the Work (as defined in the below author licence), an exclusive licence and/or a non-exclusive licence for contributions from authors who are: i) UK Crown employees; ii) where BMJ has agreed a CC-BY licence shall apply, and/or iii) in accordance with the terms applicable for US Federal Government officers or employees acting as part of their official duties; on a worldwide, perpetual, irrevocable, royalty-free basis to BMJ Publishing Group Ltd ("BMJ") its licensees and where the relevant Journal is co-owned by BMJ to the co-owners of the Journal, to publish the Work in this journal and any other BMJ products and to exploit all rights, as set out in our licence.

The Submitting Author accepts and understands that any supply made under these terms is made by BMJ to the Submitting Author unless you are acting as an employee on behalf of your employer or a postgraduate student of an affiliated institution which is paying any applicable article publishing charge ("APC") for Open Access articles. Where the Submitting Author wishes to make the Work available on an Open Access basis (and intends to pay the relevant APC), the terms of reuse of such Open Access shall be governed by a Creative Commons licence – details of these licences and which Creative Commons licence will apply to this Work are set out in our licence referred to above.

Other than as permitted in any relevant BMJ Author's Self Archiving Policies, I confirm this Work has not been accepted for publication elsewhere, is not being considered for publication elsewhere and does not duplicate material already published. I confirm all authors consent to publication of this Work and authorise the granting of this licence.

For peer review only

A single postoperative infusion of zoledronic acid to improve patient reported function after hip or knee replacement: study protocol for a randomized, controlled, double-blinded clinical trial.

For peer review only

Jonathan Brandt M.D.[1,2], Håkan Ledin M.D. [1,2], Jonas Ranstam Ph.D.[3], Ewa Roos P.T., Ph.D.[4], Per Aspenberg M.D.[2]*, Ph.D.[2] and Jörg Schilcher M.D., Ph.D.[2]

[1] Department of Orthopaedic Surgery, Capio Specialistvård Motala, Motala, Sweden

[2] Department of Orthopaedics and Department of Biomedical and Clinical Sciences, Faculty of Health Science, Linköping University, Linköping, Sweden

[3] Department of Clinical Sciences Lund, Orthopaedics, Lund University, Lund, Sweden

[4] Research Unit for Musculoskeletal Function and Physiotherapy, Institute of Sports Science and Clinical Biomechanics, University of Southern Denmark, Odense, Denmark

* Author deceased

Correspondence to
Dr Jörg Schilcher, Study Sponsor. Department of Orthopaedic Surgery, Linkoping University Hospital, 58185 Linkoping, Sweden, +46 10-103 43 12, jhschilcher@googlemail.com

Key words
Total joint replacement, Aseptic loosening, Patient reported outcome measures, Alendronate

Word count
3135

ABSTRACT

Introduction
In Sweden roughly 3000 patients are reoperated each year due to pain and loss of function related to a loosened hip or knee prosthesis. These reoperations are strenuous for the patient, technically demanding and costly for the healthcare system. Any such reoperation that can be prevented would be of great benefit. Bisphosphonates are drugs that inhibit osteoclast function. Several clinical trials suggest that bisphosphonates lead to improved implant fixation and one small study even indicates better functional outcome. Furthermore, in epidemiological studies bisphosphonates have been shown to decrease the rate of revision for aseptic loosening by half. Thus, there are several indirect indications that bisphosphonates could improve patient reported outcome, but no firm evidence.

Methods and analysis
This is a randomized, placebo controlled, double-blinded, academic clinical trial of a single postoperative dose of zoledronic acid, in patients younger than 80 years undergoing primary total hip- or knee replacement for osteoarthritis. Participants will be recruited from two orthopaedic departments. All surgeries will be performed, and study drugs given at Motala Hospital. Sweden. The primary endpoint is to investigate between-group differences in the Hip dysfunction and Osteoarthritis Outcome Score (HOOS) and the Knee injury and Osteoarthritis Outcome Score (KOOS) at three years follow-up. Secondary outcomes will be investigated at one, three and six years, and stratified for hip and knee implants. These secondary endpoints are supportive, exploratory or explanatory. A total of 1000 patients will be included in the study.

Ethics and dissemination
The study has been approved by the Regional Ethical Review Board in Linköping (DNR 2015/286-31). The study will be reported in accordance with the Consolidated Standards of Reporting Trials statement for pharmacological trials. The results will be published in peer-reviewed academic journals and disseminated to patient organisations and the media.

Registration details
The study is registered at EudraCT (No 2015-001200-55).

ARTICLE SUMMARY

Strengths and limitations of this study
- This is the first study to examine if a single intravenous dose zoledronic acid can improve patient reported outcome after primary total hip- or knee replacement.
- With 1000 patients included, this is the largest drug trial ever performed to test the effect of bisphosphonate treatment on the outcome after total joint replacement.
- The primary outcome variables HOOS and KOOS are well validated and aim to directly evaluate patient reported outcomes without the use of surrogate variables.
- Plain radiographs allow indirect assessment of treatment efficacy through radiographic evaluation of implant fixation as a secondary outcome.
- All patients are recruited from only two centres and operated at one single hospital, which might limit generalizability of the results.

For peer review only

INTRODUCTION

Total hip arthroplasty (THA) is one of the most successful operations ever invented and has been called "The operation of the century".(1) Total knee arthroplasty (TKA) has similar success rates.(2, 3) When performed in elderly patients one can expect a less than 10% chance of ever needing secondary surgery.(4, 5) However, in Sweden roughly 3000 patients are operated on annually mainly because of pain and loss of function related to a loosened hip- or knee prosthesis.(4) These reoperations are often difficult for the surgeon and patient, and the economic cost is several folds higher than for primary operation.(6) Also, results are less beneficial(7) and the complication rate is higher.(8) Any such reoperation than can be prevented would be of great benefit.

Implants loosen due to resorption of their bone bed by osteoclasts. When an implant is inserted into bone, a fracture healing response is activated.(9) This includes an increase in local bone formation and resorption, which are not necessarily coupled. If resorption outweighs bone formation the initial fixation of the implant might be impaired, leading to early subclinical loosening.(10, 11) Bisphosphonates specifically inhibit osteoclast activity, while in the fracture healing context bone formation remains increased. Therefore, bisphosphonate treatment at the time of implant insertion creates a positive balance between bone formation and resorption leading to a net anabolic effect in the bone surrounding the implant.(12) Several randomised trials have shown that bisphosphonate treatment at the time of surgery improves implant fixation in TKA,(13) THA(14, 15) and dental implants.(16) One clinical trial comprising a small sample of younger patients (n=50) also reported an improved functional outcome on the Harris Hip Score.(14) Moreover, a recent meta-analysis of four epidemiological studies using hip and knee arthroplasty registries(17-20) has shown that bisphosphonate use is associated with a 50% decrease in the need for revision surgery.(21) Despite these findings, bisphosphonate treatment is not established in routine post-operative care to improve outcome after total joint replacement (TJR).

We here describe the study protocol for a pivotal trial designed to provide final evidence for the use of intravenous bisphosphonate to improve patient reported outcome after primary THA and TKA.

METHOD AND ANALYSIS

Study design

This is a single centre, randomized, placebo-controlled, double-blinded, academic clinical trial. Participants will be recruited from two orthopaedic departments in Region Östergötland, Sweden. The main centre for recruitment is Motala Hospital (Capio Specialistvård Motala from 1 April 2019, and previously Aleris Specialistvård Motala) where roughly 85% of all patients will be recruited. The remaining 15% will be recruited from the Department of Orthopaedic Surgery at Linköping University Hospital. All patients will be referred to both orthopaedic departments based on standard health care routines in Region Östergötland. All surgeries will be performed, and study drugs given at Motala Hospital, Sweden. Patients scheduled for primary hip- or knee arthroplasty, with respect given to inclusion and exclusion criteria, will be asked to participate both at the primary outpatient visit and after phone contact with a study nurse some weeks before the scheduled surgery. The final written consent will be given on the day of surgery. All other treatment outside the study protocol described here will be according to the clinical routines of the hospital.

For peer review only - http://bmjopen.bmj.com/site/about/guidelines.xhtml

Patients

1000 patients, 500 in each group, fulfilling the eligibility criteria will be included. Table 1 lists inclusion and exclusion criteria.

Table 1. Overview of inclusion and exclusion criteria

**Inclusion criteria**	All patients eligible for primary hip or knee prosthesis for any form of osteoarthritis, between 18 to 80 years of age.
**Exclusion criteria**	Previous or present use of bisphosphonates or other antiresorptives.
	Present use of other drugs which influence bone, e.g. anti-osteoporotic agents, glucocorticoids, anti-epileptics, or use less than a year before randomization.
	Present use of nephrotoxic medication.
	Active malignant disease.
	Pregnancy and breast feeding.
	Metabolic disease (other than osteoporosis) affecting the skeleton.
	Rheumatic disease.
	Hypocalcemia as defined by local lab criteria.
	Simultaneous bilateral surgery.
	Communication problems (drug abuse, language or behavior problems).
	Creatinine clearance (GFR) <35 mL/min.
	Regular use of corticosteroids more than 5 mg dexamethasone per day.
	Atypical fracture or osteonecrosis of the jaw.
	Expected follow-op period less than 3 years (e.g. due to uncontrolled malignancy).
	Expected to require special postoperative surveillance due to increased surgical risk (e.g. for cardiac, psychiatric condition).

Randomization procedure and blinding

When found eligible, patients will be randomized to either zoledronic acid or placebo through block randomization by the study nurse on the day of surgery. Block randomization will be used to label infusion bags for drug delivery. The type of implant (hip or knee, cemented or not) will be a stratification factor in the randomization to ensure balance among these factors. All staff involved in patient care are blinded to treatment. The nurse in the postoperative care unit who is responsible for the preparation of the study drug according to the randomization list will not be blinded. However, because this person is not otherwise involved in the study, concealment of treatment allocation is not jeopardized. The content of the infusion bag will be administered to the patient on the day after surgery by a blinded nurse in the surgical ward. The randomization list will be available for unblinding in emergency situations 24 h a day at *Apoteksbolaget AB* at Linköping University Hospital.

Intervention

Patients will be randomized to receive a single postoperative infusion of zoledronic acid 4 mg/5 ml or placebo (5ml saline) on the day after surgery.

For peer review only

Study Outcomes

Rationale for the outcome measures

In previous epidemiological studies of prosthetic loosening the endpoint has been revision surgery. We will report this parameter continuously and we will use the Swedish hip and knee arthroplasty registries to capture reoperations performed outside our uptake area. Since the overall revision rate for aseptic loosening in Sweden is around 2-3% during a 10-year period, this endpoint would demand not only a very large study sample but also a long-term follow-up to get sufficient power. Also, some patients with loosening do not undergo revision surgery. They might be too old or fragile for these demanding operations. Other patients only have modest symptoms and might refrain from a demanding reoperation. Therefore, another primary outcome must be considered for reasons of feasibility. A previous study with the same treatment protocol of zoledronic acid as ours reported not only less migration in the zoledronic acid group but also a statistically significant improvement on the Harris Hip Score(14) two and three years postoperatively, despite small numbers (n=50). This study comprised only uncemented prostheses in younger patients with osteonecrosis of the femoral head. Based on these findings and because of the clinical importance, we have chosen to use patient reported scores as our primary outcome: Hip dysfunction and Osteoarthritis Outcome Score (HOOS), Swedish version LK 2.0(22) and the Knee Injury and Osteoarthritis Outcome Score (KOOS), Swedish version LK1.0.(23) Both instruments were meticulously designed with items generated in an iterative process including input from stakeholder groups comprised of patients, orthopaedists, and physical therapists. Both instruments have undergone extensive psychometric testing(24) and are recommended for evaluation of TJR by the International Consortium for Health Outcomes Measurement. These measures are free to use and have previously been shown to be highly reliable, with excellent internal consistency (Cronbach's alpha coefficient of 0.82-0.98) in samples of people undergoing THR and TKR.(25, 26)

Our primary outcome measure will be between-group differences in KOOS/HOOS from baseline until the 3-year follow-up. Based on our literature review at the time of study design and confirmed later by the *Outcomes Measures in Rheumatology*, *Workgroup Total Joint Replacement*,(27) the subscale *pain* in HOOS/KOOS will be analysed as the primary endpoint in the confirmative analysis.

Secondary endpoints are included for supportive evidence. As secondary endpoints, we will analyse between-group differences in the remaining subscales of the KOOS/HOOS from baseline to 3 years, all subscales of the KOOS/HOOS and the RAND/SF36 (Swedish version from 2013-05-21, using the 4 week recall period)(28, 29) at 1, 3 and 6 years and signs of radiographic loosening at 3 and 6 years (Table 2). The RAND/SF36 will be analysed using physical and mental component scores.

For peer review only

Table 2. Schedule of assessments and events.

**Visit**	1	2	3	4	5	6	7	8
	Screening	Surgery	Treatment	Discharge	Follow-up	Follow-up	Follow-up	Follow-up
**Time**	Day -28 to - 2	Day 1	Day 2	Day 3-5	6 weeks	1 year ±1 month	3 year ±1 month	6 year ±1 month
**Assessment /event**								
Informed Consent*[1]	X							
Demography Medical history	X							
Physical Examination	X							
Height and Weight	X							
Assessment of inclusion and exclusion criteria	X	X						
Blood sampling	X		X					
Start of continuous daily Calcium*[2]			X					
Surgery		X						
Randomization*[3]		X	X					
Administration of IMP/Placebo*[4]			X					
HOOS/KOOS RAND/SF-36 questionnaire	X					X	X	X
X-ray		X					X	X
Concomitant Medication	X	X						
Routine follow-up (via phone)						X		
AE Assessment*[5]			X	X	X	X	X	X

IMP = investigational medical product, HOOS = Hip dysfunction and Osteoarthritis Outcome Score, KOOS = Knee injury and Osteoarthritis Outcome Score, SF-36 = 36 item Short Form, X-ray = Plain radiography, AE = Adverse Event, SAE = Severe Adverse Event.

*[1] The Informed Consent Form must be signed before any study related procedure

*[2] Vitamin D and calcium will be given daily in standard dosage from day 2 for 1 month.

*[3] Randomization has to be performed as close as possible prior to the first IMP infusion

*[4] Administration of IMP/Placebo will be given the day after surgery.

For peer review only

*[5] AE/SAE will be collected via a questionnaire at visit 5 and personal interview at 4. At visit 6, 7 and 8 only SAE will be collected via a questionnaire. Reminders will be given by phone.

Statistical analysis

Power

Both HOOS and KOOS ranges from 0 to 100. The minimal important change is often reported to be 8-10 points but this estimate is dependent on contextual factors such as patient age, intervention and time to follow-up, and according to the developers of KOOS (www.koos.nu) no generic value of the minimal important change is available for KOOS, or any other patient reported outcome measure. In a large sample a small statistically significant difference is still indicative of an important treatment effect, even if the difference is smaller than the minimal clinically important difference. At the time of the study design average reported KOOS values for 3 years after TKR were not available, and sample size calculations therefore were based on average values 2 years after TKR: 84, (SD 14). These values would with Student's t-test and a two-sided significance level of 5 % yield 90 % power to show a difference of 3 points in KOOS/HOOS with 450 patients in each arm. To compensate for a 10% withdrawal, a further 50 patients would be needed, leading to a total of 1000 patients to include in a superiority trial.

Statistical analysis plan

Statistical analysis of the primary endpoint will be carried out using a mixed model repeated measurements ANOVA of the changes in KOOS/HOOS from baseline until 3-years follow-up with covariate adjustment for baseline values of HOOS/KOOS, and with implant type (hip, knee and cemented, uncemented) and age (continuous) as further covariates. As supportive endpoints, we will analyse HOOS/KOOS subscales at 6-years follow-up, signs of radiographic loosening at 3 and 6 years and SF/RAND36 at 1, 3 and 6 years. we will also perform subgroup analysis of men and women and implant types.

To reduce the risk of bias during interpretation, blinded results from the analyses (with study groups labelled as group A and group B) will be presented to all the authors, who will agree in writing on two alternative interpretations.(30) Thereafter, the data manager will break the randomization code.

As part of an exploratory analysis and to be able to define the clinical impact of our results we will perform a responder analysis comparing the proportion of patients who achieve a substantial clinical improvement on the subscale *pain* in HOOS/KOOS between the treatment and the control group.(31) HOOS and KOOS values depend on age, BMI and sex, which will be considered as cofactors in the analysis.

No interim analysis will be performed.

Safety

Concomitant drug treatment

After the infusion, oral supplements of vitamin D and calcium will be given once daily to both groups for the first postoperative month to prevent bisphosphonate-induced hypocalcaemia.

For peer review only - http://bmjopen.bmj.com/site/about/guidelines.xhtml

Zoledronic acid

Repeated infusions of zoledronic acid has been associated with a slight increase in atrial fibrillation in the highest age groups(32) and because of this we have set an upper age limit for inclusion to 80 years. Bisphosphonate use is strongly associated with osteonecrosis of the jaw. This is however a very rare condition and only associated with multiple dosing over time.(33) 10% of patients treated with zoledronic acid have reported influenza-like symptoms(34) which can lead to a prolonged hospital stay. Even though zoledronic acid has not been reported to cause hypocalcaemia in osteoporosis treatment,(35) normal preoperative calcium and vitamin D levels are required for inclusion for safety reasons and patients will get oral supplements for the first postoperative month. Zoledronic acid can affect kidney function and the manufacturer recommends against its use in patients with creatinine clearance <35 ml/min.(36) Bisphosphonates are used in large scale to treat patients with osteoporosis, and its safety is extensively documented.(33) Furthermore, in this study only one dose is given.

Adverse events

Adverse events (AE) are followed for 6 weeks after the infusion and are recorded at the 6-week follow-up at the physiotherapist. After that AE's will be collected annually until the end of the study. An AE is defined in the International Conference on Harmonization (ICH) Guidelines for Good Clinical Practice as "any untoward medial occurrence in a patient or clinical investigation subject administered a pharmaceutical product and does not necessarily have a causal relationship with this treatment". The occurrence of atypical femoral fractures and osteonecrosis of the jaw will be recorded throughout the whole study period. Serious adverse event (SAE) is defined as an AE that is fatal, life threatening, requires in-patient hospitalization or prolonged hospitalization, results in persistent or significant disability/incapacity or other significant medical hazards. Adverse Drug Reaction (ADR) is defined as all untoward and unintended response to a medical product related to any dose administered and will also be recorded during the first 6 weeks.
The occurrence of AE and SAE will be presented descriptively.

ETHICS AND DISSEMINATION

Ethics

The study will be conducted in agreement with the Helsinki declaration and ICH guidelines will be adhered to. The study will be monitored by *Forum Östergötland,* which is part of the national organisation for Clinical Studies in Sweden, Forum Sydost. All completed questionnaires will be kept secured from unauthorized access within the research nurses´ facility. Data for the purpose of statistical analyses will be collected in digitized files. Other data will be stored in the patients´ ordinary medical chart. The study has been approved by the Regional Ethical Review Board in Linköping (DNR 2015/286-31).

Dissemination

The results of this study will be published in an international peer reviewed scientific paper regardless of whether the results are positive, negative or inconclusive regarding the hypothesis of the study.

Patient and Public Involvement

No patient organisation or patient representatives were involved in the design of the study. The results of our study will be disseminated to patient organizations and the public through the Swedish Orthopaedic Association and the Swedish National Joint Arthroplasty Register.

For peer review only

DISCUSSION

Strengths and limitations

Strengths

The main strength of this study is its size and design. To our knowledge this is the largest RCT designed to elucidate if bisphosphonates can improve outcome in primary THA and TKA. If we can demonstrate a significant increase in patient satisfaction after bisphosphonate administration, it could revolutionize the operative care of patients undergoing TJR.

Limitations

The main limitation of this study can be considered its primary patient-reported outcome measure. A hard endpoint as a prospectively collected rate of revision would be preferable, however not feasible in this research question. Also, not all patients with loosening of their implants undergo revision surgery. Some are too old and fragile, and others have moderate symptoms and might decide to abstain from surgery. The use of a patient-reported primary endpoint will increase the relevance of the findings to patients. Improved patient reported outcomes after TJR might in fact be more important for the majority of the patients undergoing TJR compared to prosthetic loosening assessed on radiographs, which is a secondary outcome. If we fail to demonstrate a significant increase in patient reported outcomes, we might be able to show a decrease in radiographic signs of early loosening, which strongly correlates with late aseptic loosening.(37, 38)

Authors affiliation

1 Department of Orthopaedic Surgery, Capio Specialistvård Motala, Motala, Sweden

2 Department of Orthopaedics and Experimental and Clinical Medicine, Faculty of Health Science, Linköping University, Linköping, Sweden

3 Department of Clinical Sciences Lund, Orthopaedics, Lund University, Lund, Sweden

4 Research Unit for Musculoskeletal Function and Physiotherapy, Institute of Sports Science and Clinical Biomechanics, University of Southern Denmark, Odense, Denmark

Author contributions

JB: Writing of manuscript, study design
HL: Writing of manuscript, study design
JR: Revision of manuscript, study design
ER: Revision of manuscript, study design
PA: Study design, preparation of manuscript
JS: Writing and revision of manuscript, study design

Funding statement

This academic drug trial is run without any support from the industry. Financial support was received from the Swedish Research Council and ALF-grants from Region Östergötland, Sweden.

Data statement

The dataset will be made available in a data repository.

For peer review only

Competing interests

None stated. Data collection, management, analysis, and interpretation of data; writing of the report; and the decision to submit the report for publication are the sole authority of the study sponsor.

Acknowledgements

We are grateful for the research-friendly leadership of the healthcare providers Capio Specialistvård Motala and Aleris Specialistvård Motala. We also thank all personnel at Motala hospital for their extra effort to make this large research project possible, in specific our research nurses Anneli Kvarnestedt and Carmen Henriksson.

REFERENCES

1. Learmonth ID, Young C, Rorabeck C. The operation of the century: total hip replacement. *Lancet* 2007;370:1508-19.
2. Evans JT, Walker RW, Evans JP, et al. How long does a knee replacement last? A systematic review and meta-analysis of case series and national registry reports with more than 15 years of follow-up. *The Lancet* 2019;393:655-63.
3. Beswick AD, Wylde V, Gooberman-Hill R, et al. What proportion of patients report long-term pain after total hip or knee replacement for osteoarthritis? A systematic review of prospective studies in unselected patients. *BMJ Open* 2012;2:e000435.
4. Kärrholm J MM, Odin M, Vinblad J, Rogmark C, Rolfson O. The Swedish Hip Arthroplasty Register, Annual Report 2017. 2018.
5. Robertsson O LL, Sundberg M, W-Dahl A. The Swedish Knee Arthroplasty Register, Annual Report 2019. 2019.
6. Vanhegan IS, Malik AK, Jayakumar P, et al. A financial analysis of revision hip arthroplasty: the economic burden in relation to the national tariff. *J Bone Joint Surg Br* 2012;94:619-23.
7. Lenguerrand E, Whitehouse MR, Wylde V, et al. Pain and Function Recovery Trajectories following Revision Hip Arthroplasty: Short-Term Changes and Comparison with Primary Hip Arthroplasty in the ADAPT Cohort Study. *PLoS One* 2016;11:e0164839.
8. Ong KL, Lau E, Suggs J, et al. Risk of subsequent revision after primary and revision total joint arthroplasty. *Clin Orthop Relat Res* 2010;468:3070-6.
9. Bernhardsson M, Sandberg O, Aspenberg P. Experimental models for cancellous bone healing in the rat. *Acta Orthop* 2015;86:745-50.
10. Schindeler A, McDonald MM, Bokko P, et al. Bone remodeling during fracture repair: The cellular picture. *Semin Cell Dev Biol* 2008;19:459-66.
11. Agholme F, Aspenberg P. Experimental results of combining bisphosphonates with allograft in a rat model. *J Bone Joint Surg Br* 2009;91:670-5.
12. Yu NY, Schindeler A, Tagil M, et al. Use of BMPs and bisphosphonates in improving bone fracture healing. *Front Biosci (Elite Ed)* 2012;4:2647-53.
13. Hilding M, Aspenberg P. Local peroperative treatment with a bisphosphonate improves the fixation of total knee prostheses: a randomized, double-blind radiostereometric study of 50 patients. *Acta Orthop* 2007;78:795-9.
14. Friedl G, Radl R, Stihsen C, et al. The effect of a single infusion of zoledronic acid on early implant migration in total hip arthroplasty. A randomized, double-blind, controlled trial. *J Bone Joint Surg Am* 2009;91:274-81.
15. Schilcher J, Palm L, Ivarsson I, et al. Local bisphosphonate reduces migration and formation of radiolucent lines adjacent to cemented acetabular components. *Bone Joint J* 2017;99-B:317-24.
16. Abtahi J, Tengvall P, Aspenberg P. A bisphosphonate-coating improves the fixation of metal implants in human bone. A randomized trial of dental implants. *Bone* 2012;50:1148-51.

For peer review only

17. Thillemann TM, Pedersen AB, Mehnert F, et al. Postoperative use of bisphosphonates and risk of revision after primary total hip arthroplasty: a nationwide population-based study. *Bone* 2010;46:946-51.
18. Prieto-Alhambra D, Javaid MK, Judge A, et al. Association between bisphosphonate use and implant survival after primary total arthroplasty of the knee or hip: population based retrospective cohort study. *BMJ* 2011;343:d7222.
19. Prieto-Alhambra D, Lalmohamed A, Abrahamsen B, et al. Oral bisphosphonate use and total knee/hip implant survival: validation of results in an external population-based cohort. *Arthritis Rheumatol* 2014;66:3233-40.
20. Khatod M, Inacio MC, Dell RM, et al. Association of Bisphosphonate Use and Risk of Revision After THA: Outcomes From a US Total Joint Replacement Registry. *Clin Orthop Relat Res* 2015;473:3412-20.
21. Teng S, Yi C, Krettek C, et al. Bisphosphonate Use and Risk of Implant Revision after Total Hip/Knee Arthroplasty: A Meta-Analysis of Observational Studies. *PLoS One* 2015;10:e0139927.
22. Nilsdotter AK, Lohmander LS, Klassbo M, et al. Hip disability and osteoarthritis outcome score (HOOS)--validity and responsiveness in total hip replacement. *BMC Musculoskelet Disord* 2003;4:10.
23. Roos EM, Toksvig-Larsen S. Knee injury and Osteoarthritis Outcome Score (KOOS) - validation and comparison to the WOMAC in total knee replacement. *Health Qual Life Outcomes* 2003;1:17.
24. Losina E, Ranstam J, Collins JE, et al. OARSI Clinical Trials Recommendations: Key analytic considerations in design, analysis, and reporting of randomized controlled trials in osteoarthritis. *Osteoarthritis Cartilage* 2015;23:677-85.
25. Nilsdotter A, Bremander A. Measures of hip function and symptoms: Harris Hip Score (HHS), Hip Disability and Osteoarthritis Outcome Score (HOOS), Oxford Hip Score (OHS), Lequesne Index of Severity for Osteoarthritis of the Hip (LISOH), and American Academy of Orthopedic Surgeons (AAOS) Hip and Knee Questionnaire. *Arthritis Care Res (Hoboken)* 2011;63 Suppl 11:S200-7.
26. Collins NJ, Roos EM. Patient-reported outcomes for total hip and knee arthroplasty: commonly used instruments and attributes of a "good" measure. *Clin Geriatr Med* 2012;28:367-94.
27. Singh JA, Dowsey MM, Dohm M, et al. Achieving Consensus on Total Joint Replacement Trial Outcome Reporting Using the OMERACT Filter: Endorsement of the Final Core Domain Set for Total Hip and Total Knee Replacement Trials for Endstage Arthritis. *J Rheumatol* 2017;44:1723-6.
28. Hays RD, Sherbourne CD, Mazel RM. The RAND 36-Item Health Survey 1.0. *Health Econ* 1993;2:217-27.
29. Ware JE, Jr., Gandek B. Overview of the SF-36 Health Survey and the International Quality of Life Assessment (IQOLA) Project. *J Clin Epidemiol* 1998;51:903-12.
30. Jarvinen TL, Sihvonen R, Bhandari M, et al. Blinded interpretation of study results can feasibly and effectively diminish interpretation bias. *J Clin Epidemiol* 2014;67:769-72.
31. Lyman S, Lee YY, McLawhorn AS, et al. What Are the Minimal and Substantial Improvements in the HOOS and KOOS and JR Versions After Total Joint Replacement? *Clin Orthop Relat Res* 2018;476:2432-41.
32. Kim DH, Rogers JR, Fulchino LA, et al. Bisphosphonates and risk of cardiovascular events: a meta-analysis. *PLoS One* 2015;10:e0122646.
33. Reyes C, Hitz M, Prieto-Alhambra D, et al. Risks and Benefits of Bisphosphonate Therapies. *J Cell Biochem* 2016;117:20-8.
34. Kotian P, Boloor A, Sreenivasan S. Study of Adverse Effect Profile of Parenteral Zoledronic Acid in Female Patients with Osteoporosis. *J Clin Diagn Res* 2016;10:OC04-6.
35. Kreutle V, Blum C, Meier C, et al. Bisphosphonate induced hypocalcaemia - report of six cases and review of the literature. *Swiss Med Wkly* 2014;144:w13979.
36. McClung M, Harris ST, Miller PD, et al. Bisphosphonate therapy for osteoporosis: benefits, risks, and drug holiday. *Am J Med* 2013;126:13-20.

For peer review only

37. Pijls BG, Nieuwenhuijse MJ, Fiocco M, et al. Early proximal migration of cups is associated with late revision in THA: a systematic review and meta-analysis of 26 RSA studies and 49 survivalstudies. *Acta Orthop* 2012;83:583-91.
38. Pijls BG, Valstar ER, Nouta KA, et al. Early migration of tibial components is associated with late revision: a systematic review and meta-analysis of 21,000 knee arthroplasties. *Acta Orthop* 2012;83:614-24.

For peer review only

Reporting checklist for protocol of a clinical trial.

Based on the SPIRIT guidelines.

Instructions to authors

Complete this checklist by entering the page numbers from your manuscript where readers will find each of the items listed below.

Your article may not currently address all the items on the checklist. Please modify your text to include the missing information. If you are certain that an item does not apply, please write "n/a" and provide a short explanation.

Upload your completed checklist as an extra file when you submit to a journal.

In your methods section, say that you used the SPIRITreporting guidelines, and cite them as:

Chan A-W, Tetzlaff JM, Altman DG, Laupacis A, Gøtzsche PC, Krleža-Jerić K, Hróbjartsson A, Mann H, Dickersin K, Berlin J, Doré C, Parulekar W, Summerskill W, Groves T, Schulz K, Sox H, Rockhold FW, Rennie D, Moher D. SPIRIT 2013 Statement: Defining standard protocol items for clinical trials. Ann Intern Med. 2013;158(3):200-207

For peer review only

		Reporting Item	Page Number
**Administrative information**			
Title	#1	Descriptive title identifying the study design, population, interventions, and, if applicable, trial acronym	1

Trial registration	#2a	Trial identifier and registry name. If not yet registered, name of intended registry	2
Trial registration: data set	#2b	All items from the World Health Organization Trial Registration Data Set	2
Protocol version	#3	Date and version identifier	2
Funding	#4	Sources and types of financial, material, and other support	10
Roles and responsibilities: contributorship	#5a	Names, affiliations, and roles of protocol contributors	9
Roles and responsibilities: sponsor contact information	#5b	Name and contact information for the trial sponsor	1
Roles and responsibilities: sponsor and funder	#5c	Role of study sponsor and funders, if any, in study design; collection, management, analysis, and interpretation of data; writing of the report; and the decision to submit the report for publication, including whether they will have ultimate authority over any of these activities	9
Roles and responsibilities: committees	#5d	Composition, roles, and responsibilities of the coordinating centre, steering committee, endpoint adjudication committee, data management team, and	-

For peer review only

		other individuals or groups overseeing the trial, if applicable (see Item 21a for data monitoring committee)	
**Introduction**			
Background and rationale	#6a	Description of research question and justification for undertaking the trial, including summary of relevant studies (published and unpublished) examining benefits and harms for each intervention	3
Background and rationale: choice of comparators	#6b	Explanation for choice of comparators	5
Objectives	#7	Specific objectives or hypotheses	3
Trial design	#8	Description of trial design including type of trial (eg, parallel group, crossover, factorial, single group), allocation ratio, and framework (eg, superiority, equivalence, non-inferiority, exploratory)	3
**Methods: Participants, interventions, and outcomes**			
Study setting	#9	Description of study settings (eg, community clinic, academic hospital) and list of countries where data will be collected. Reference to where list of study sites can be obtained	3

For peer review only

Eligibility criteria	#10	Inclusion and exclusion criteria for participants. If applicable, eligibility criteria for study centres and individuals who will perform the interventions (eg, surgeons, psychotherapists)	4
Interventions: description	#11a	Interventions for each group with sufficient detail to allow replication, including how and when they will be administered	4
Interventions: modifications	#11b	Criteria for discontinuing or modifying allocated interventions for a given trial participant (eg, drug dose change in response to harms, participant request, or improving / worsening disease)	-
Interventions: adherance	#11c	Strategies to improve adherence to intervention protocols, and any procedures for monitoring adherence (eg, drug tablet return; laboratory tests)	6
Interventions: concomitant care	#11d	Relevant concomitant care and interventions that are permitted or prohibited during the trial	4
Outcomes	#12	Primary, secondary, and other outcomes, including the specific measurement variable (eg, systolic blood pressure), analysis metric (eg, change from baseline, final value, time to event), method of aggregation (eg, median, proportion), and time point for each outcome. Explanation of the clinical relevance of chosen efficacy and harm outcomes is strongly recommended	5

For peer review only

Participant timeline	#13	Time schedule of enrolment, interventions (including any run-ins and washouts), assessments, and visits for participants. A schematic diagram is highly recommended (see Figure)	6
Sample size	#14	Estimated number of participants needed to achieve study objectives and how it was determined, including clinical and statistical assumptions supporting any sample size calculations	7
Recruitment	#15	Strategies for achieving adequate participant enrolment to reach target sample size	4
**Methods: Assignment of interventions (for controlled trials)**			
Allocation: sequence generation	#16a	Method of generating the allocation sequence (eg, computer-generated random numbers), and list of any factors for stratification. To reduce predictability of a random sequence, details of any planned restriction (eg, blocking) should be provided in a separate document that is unavailable to those who enrol participants or assign interventions	4
Allocation concealment mechanism	#16b	Mechanism of implementing the allocation sequence (eg, central telephone; sequentially numbered, opaque,	4

For peer review only

		sealed envelopes), describing any steps to conceal the sequence until interventions are assigned	
Allocation: implementation	#16c	Who will generate the allocation sequence, who will enrol participants, and who will assign participants to interventions	4
Blinding (masking)	#17a	Who will be blinded after assignment to interventions (eg, trial participants, care providers, outcome assessors, data analysts), and how	4
Blinding (masking): emergency unblinding	#17b	If blinded, circumstances under which unblinding is permissible, and procedure for revealing a participant's allocated intervention during the trial	4
**Methods: Data collection, management, and analysis**			
Data collection plan	#18a	Plans for assessment and collection of outcome, baseline, and other trial data, including any related processes to promote data quality (eg, duplicate measurements, training of assessors) and a description of study instruments (eg, questionnaires, laboratory tests) along with their reliability and validity, if known. Reference to where data collection forms can be found, if not in the protocol	6

For peer review only

Data collection plan: retention	#18b	Plans to promote participant retention and complete follow-up, including list of any outcome data to be collected for participants who discontinue or deviate from intervention protocols	6
Data management	#19	Plans for data entry, coding, security, and storage, including any related processes to promote data quality (eg, double data entry; range checks for data values). Reference to where details of data management procedures can be found, if not in the protocol	9
Statistics: outcomes	#20a	Statistical methods for analysing primary and secondary outcomes. Reference to where other details of the statistical analysis plan can be found, if not in the protocol	5
Statistics: additional analyses	#20b	Methods for any additional analyses (eg, subgroup and adjusted analyses)	5
Statistics: analysis population and missing data	#20c	Definition of analysis population relating to protocol non-adherence (eg, as randomised analysis), and any statistical methods to handle missing data (eg, multiple imputation)	5

Methods: Monitoring

Data monitoring: formal committee	#21a	Composition of data monitoring committee (DMC); summary of its role and reporting structure; statement of whether it is independent from the sponsor and competing interests; and reference to where further	8

For peer review only

		details about its charter can be found, if not in the protocol. Alternatively, an explanation of why a DMC is not needed	
Data monitoring: interim analysis	#21b	Description of any interim analyses and stopping guidelines, including who will have access to these interim results and make the final decision to terminate the trial	7
Harms	#22	Plans for collecting, assessing, reporting, and managing solicited and spontaneously reported adverse events and other unintended effects of trial interventions or trial conduct	8
Auditing	#23	Frequency and procedures for auditing trial conduct, if any, and whether the process will be independent from investigators and the sponsor	8
**Ethics and dissemination**			
Research ethics approval	#24	Plans for seeking research ethics committee / institutional review board (REC / IRB) approval	8
Protocol amendments	#25	Plans for communicating important protocol modifications (eg, changes to eligibility criteria, outcomes, analyses) to relevant parties (eg, investigators, REC / IRBs, trial participants, trial 8registries, journals, regulators)	8

For peer review only

Consent or assent	#26a	Who will obtain informed consent or assent from potential trial participants or authorised surrogates, and how (see Item 32)	3
Consent or assent: ancillary studies	#26b	Additional consent provisions for collection and use of participant data and biological specimens in ancillary studies, if applicable	
Confidentiality	#27	How personal information about potential and enrolled participants will be collected, shared, and maintained in order to protect confidentiality before, during, and after the trial	8
Declaration of interests	#28	Financial and other competing interests for principal investigators for the overall trial and each study site	9
Data access	#29	Statement of who will have access to the final trial dataset, and disclosure of contractual agreements that limit such access for investigators	9
Ancillary and post trial care	#30	Provisions, if any, for ancillary and post-trial care, and for compensation to those who suffer harm from trial participation	-
Dissemination policy: trial results	#31a	Plans for investigators and sponsor to communicate trial results to participants, healthcare professionals, the public, and other relevant groups (eg, via publication, reporting in results databases, or other data sharing arrangements), including any publication restrictions	9

For peer review only

Dissemination policy: authorship	#31b	Authorship eligibility guidelines and any intended use of professional writers	9
Dissemination policy: reproducible research	#31c	Plans, if any, for granting public access to the full protocol, participant-level dataset, and statistical code	9

Appendices

Informed consent materials	#32	Model consent form and other related documentation given to participants and authorised surrogates	-
Biological specimens	#33	Plans for collection, laboratory evaluation, and storage of biological specimens for genetic or molecular analysis in the current trial and for future use in ancillary studies, if applicable	-

None The SPIRIT checklist is distributed under the terms of the Creative Commons Attribution License CC-BY-ND 3.0. This checklist can be completed online using https://www.goodreports.org/, a tool made by the EQUATOR Network in collaboration with Penelope.ai

For peer review only

BMJ Open

A single postoperative infusion of zoledronic acid to improve patient reported outcome after hip or knee replacement: study protocol for a randomized, controlled, double-blinded clinical trial.

Journal:	*BMJ Open*
Manuscript ID	bmjopen-2020-040985.R1
Article Type:	Protocol
Date Submitted by the Author:	02-Aug-2020
Complete List of Authors:	Brandt, Jonathan; Capio Specialistvård Motala, Department of Orthopaedic Surgery<br>Ledin, Håkan; Capio Specialistvård Motala, Department of Orthopaedic Surgery<br>Ranstam, Jonas; Lunds Universitet, Clinical sciences<br>Roos, Ewa; Syddansk Universitet Det Sundhedsvidenskabelige Fakultet, Sports Science and Clinical Biomechanics<br>Aspenberg, Per; Linköping University Hospital, Department of Orthopaedics in Linköping, and Department of Biomedical and Clinical Sciences, Linköping University<br>Schilcher, Jörg; Linköping University Hospital, Department of Orthopaedics and Department of Biomedical and Clinical Sciences, Faculty of Health Science
**Primary Subject Heading**:	Surgery
Secondary Subject Heading:	Complementary medicine
Keywords:	Adult orthopaedics < ORTHOPAEDIC & TRAUMA SURGERY, Hip < ORTHOPAEDIC & TRAUMA SURGERY, Knee < ORTHOPAEDIC & TRAUMA SURGERY

SCHOLARONE™
Manuscripts

I, the Submitting Author has the right to grant and does grant on behalf of all authors of the Work (as defined in the below author licence), an exclusive licence and/or a non-exclusive licence for contributions from authors who are: i) UK Crown employees; ii) where BMJ has agreed a CC-BY licence shall apply, and/or iii) in accordance with the terms applicable for US Federal Government officers or employees acting as part of their official duties; on a worldwide, perpetual, irrevocable, royalty-free basis to BMJ Publishing Group Ltd ("BMJ") its licensees and where the relevant Journal is co-owned by BMJ to the co-owners of the Journal, to publish the Work in this journal and any other BMJ products and to exploit all rights, as set out in our licence.

The Submitting Author accepts and understands that any supply made under these terms is made by BMJ to the Submitting Author unless you are acting as an employee on behalf of your employer or a postgraduate student of an affiliated institution which is paying any applicable article publishing charge ("APC") for Open Access articles. Where the Submitting Author wishes to make the Work available on an Open Access basis (and intends to pay the relevant APC), the terms of reuse of such Open Access shall be governed by a Creative Commons licence – details of these licences and which Creative Commons licence will apply to this Work are set out in our licence referred to above.

Other than as permitted in any relevant BMJ Author's Self Archiving Policies, I confirm this Work has not been accepted for publication elsewhere, is not being considered for publication elsewhere and does not duplicate material already published. I confirm all authors consent to publication of this Work and authorise the granting of this licence.

For peer review only

A single postoperative infusion of zoledronic acid to improve patient reported outcome after hip or knee replacement: study protocol for a randomized, controlled, double-blinded clinical trial.

For peer review only

Jonathan Brandt M.D.[1,2], Håkan Ledin M.D. [1,2], Jonas Ranstam Ph.D.[3], Ewa Roos P.T., Ph.D.[4], Per Aspenberg M.D.[2]*, Ph.D.[2] and Jörg Schilcher M.D., Ph.D.[2]

[1] Department of Orthopaedic Surgery, Capio Specialistvård Motala, Motala, Sweden

[2] Department of Orthopaedics and Department of Biomedical and Clinical Sciences, Faculty of Health Science, Linköping University, Linköping, Sweden

[3] Department of Clinical Sciences Lund, Orthopaedics, Lund University, Lund, Sweden

[4] Research Unit for Musculoskeletal Function and Physiotherapy, Institute of Sports Science and Clinical Biomechanics, University of Southern Denmark, Odense, Denmark

[*] Author deceased

Correspondence to
Dr Jörg Schilcher, Study Sponsor. Department of Orthopaedic Surgery, Linköping University Hospital, 58185 Linköping, Sweden, +46 10-103 43 12, jorg.schilcher@liu.se

Key words
Total joint replacement, Aseptic loosening, Patient reported outcome measures, Zoledronate

Word count
3273

ABSTRACT

Introduction

In Sweden roughly 3000 patients are reoperated each year due to pain and loss of function related to a loosened hip or knee prosthesis. These reoperations are strenuous for the patient, technically demanding and costly for the healthcare system. Any such reoperation that can be prevented would be of great benefit. Bisphosphonates are drugs that inhibit osteoclast function. Several clinical trials suggest that bisphosphonates lead to improved implant fixation and one small study even indicates better functional outcome. Furthermore, in epidemiological studies bisphosphonates have been shown to decrease the rate of revision for aseptic loosening by half. Thus, there are several indirect indications that bisphosphonates could improve patient reported outcome, but no firm evidence.

Methods and analysis

This is a pragmatic randomized, placebo-controlled, double-blinded, academic clinical trial of a single postoperative dose of zoledronic acid, in patients younger than 80 years undergoing primary total hip- or knee replacement for osteoarthritis. Participants will be recruited from two orthopaedic departments. All surgeries will be performed, and study drugs given at Motala Hospital. Sweden. The primary endpoint is to investigate between-group differences in the Hip dysfunction and Osteoarthritis Outcome Score (HOOS) and the Knee injury and Osteoarthritis Outcome Score (KOOS) at three years follow-up. Secondary outcomes will be investigated at one, three and six years, and stratified for hip and knee implants. These secondary endpoints are supportive, exploratory or explanatory. A total of 1000 patients will be included in the study.

Ethics and dissemination

The study has been approved by the Regional Ethical Review Board in Linköping (DNR 2015/286-31). The study will be reported in accordance with the Consolidated Standards of Reporting Trials statement for pharmacological trials. The results will be submitted for publication in peer-reviewed academic journals and disseminated to patient organisations and the media.

Registration details

The study is registered at EudraCT (No 2015-001200-55).

ARTICLE SUMMARY

Strengths and limitations of this study

- This is the first study to examine if a single intravenous dose zoledronic acid can improve patient reported outcome after primary total hip- or knee replacement.
- With 1000 patients included, this is the largest drug trial ever performed to test the effect of bisphosphonate treatment on the outcome after total joint replacement.
- The primary outcome variables HOOS and KOOS are well validated and aim to directly evaluate patient reported outcomes without the use of surrogate variables.
- Plain radiographs allow indirect assessment of treatment efficacy through radiographic evaluation of implant fixation as a secondary outcome.
- All patients are recruited from only two centres and operated at one single hospital, which might limit generalizability of the results.

For peer review only

INTRODUCTION

Total hip arthroplasty (THA) is one of the most successful operations ever invented and has been called "The operation of the century".(1) Total knee arthroplasty (TKA) has similar success rates.(2, 3) When performed in elderly patients one can expect a less than 10% chance of ever needing secondary surgery.(4, 5) However, in Sweden roughly 3000 patients are operated on annually mainly because of pain and loss of function related to a loosened hip- or knee prosthesis.(4) These reoperations are often difficult for the surgeon and the patient, and the economic cost is several folds higher than for primary operation.(6) Also, results are less beneficial(7) and the complication rate is higher.(8) Any such reoperation that can be prevented would be of great benefit.

Implants loosen due to resorption of their bone bed by osteoclasts. When an implant is inserted into bone, a fracture healing response is activated.(9) This includes an increase in local bone formation and resorption, which are not necessarily coupled. If resorption outweighs bone formation the initial fixation of the implant might be impaired. This excessive motion between the implant and its surrounding bone bed (implant migration) might allow pressurized fluid flows and invasion of wear debris particles leading to further bone resorption.(10) When direct bone contact does not occur in the early postoperative period a fibrous tissue membrane will be formed leading to early subclinical loosening.(11, 12) The primary postoperative result, i.e. the fixation, can be estimated by specific radiographic methods (radiostereometry) to measure implant migration. There is a strong correlation between postoperative migration measured with radiostereometry and late loosening, showing that the early fixation is important for the late results. For acetabular cups the area under the receiver operating characteristic curve (ROC) for increased migration 2 years postoperatively to predict loosening after 10 years is 0.88 (95% confidence interval, 0.74 to 1.00).(13) Similar associations can be found for tibial components in total knee replacement.(14) This suggests that late loosening is the final result of a continuous process that starts immediately after the operation. Radiostereometry is partly invasive and very costly and can only be used in small series of patients.

Bisphosphonates specifically inhibit osteoclast activity, while in the fracture healing context bone formation remains increased. Therefore, bisphosphonate treatment at the time of implant insertion would possibly create a positive balance between bone formation and resorption leading to a net anabolic effect in the bone surrounding the implant thus leading to a more stable primary fixation.(15) Several randomised trials have shown that bisphosphonate treatment at the time of surgery reduces implant migration in TKA,(16) THA(17, 18) and dental implants.(19) One clinical trial comprising a small sample of younger patients (n=50) also reported an improved functional outcome on the Harris Hip Score.(17) All other RCT´s showed no effect of bisphosphonate treatment on patient reported outcome, but none of these trials were powered to detect such a difference. A recent meta-analysis of four epidemiological studies using hip and knee arthroplasty registries(20-23) has shown that bisphosphonate use is associated with a 50% decrease in the need for revision surgery.(24) Despite these findings, bisphosphonate treatment is not established in routine post-operative care to improve outcome after total joint replacement (TJR).

We here describe the study protocol for a pivotal trial designed to provide final evidence for the use of intravenous bisphosphonate to improve patient reported outcome after primary THA and TKA.

For peer review only - http://bmjopen.bmj.com/site/about/guidelines.xhtml

METHOD AND ANALYSIS

Study design

This is a single centre, pragmatic, randomized, placebo-controlled, double-blinded, academic clinical trial. Participants will be recruited from two orthopaedic departments in Region Östergötland, Sweden. The main centre for recruitment is Motala Hospital (Capio Specialistvård Motala from 1 April 2019, and previously Aleris Specialistvård Motala) where roughly 85% of all patients will be recruited. The remaining 15% will be recruited from the Department of Orthopaedic Surgery at Linköping University Hospital. All patients will be referred to both orthopaedic departments based on standard health care routines in Region Östergötland. All surgeries will be performed, and study drugs given at Motala Hospital, Sweden. Patients scheduled for primary hip- or knee arthroplasty, with respect given to inclusion and exclusion criteria, will be asked to participate both at the primary outpatient visit and after phone contact with a study nurse some weeks before the scheduled surgery. The final written consent will be given on the day of surgery (supplementary file 1). All other treatment outside the study protocol described here will be according to the clinical routines of the hospital. Inclusion of patients was started January 4th, 2016. Data collection for the primary outcome will continue until patients have been followed for three years, roughly until 2024.

Patients

1000 patients, 500 in each group, fulfilling the eligibility criteria (Table 1) will be included.

Table 1. Overview of inclusion and exclusion criteria

**Inclusion criteria**	All patients eligible for primary hip or knee prosthesis for any form of osteoarthritis, between 18 to 80 years of age.
**Exclusion criteria**	Previous or present use of bisphosphonates or other antiresorptives.
	Present use of other drugs which influence bone, e.g. anti-osteoporotic agents, glucocorticoids, anti-epileptics, or use less than a year before randomization.
	Present use of nephrotoxic medication.
	Active malignant disease.
	Pregnancy and breast feeding.
	Metabolic disease (other than osteoporosis) affecting the skeleton.
	Rheumatic disease.
	Hypocalcaemia as defined by local lab criteria.
	Simultaneous bilateral surgery.
	Communication problems (drug abuse, language or behaviour problems).
	Creatinine clearance (GFR) <35 mL/min.
	Regular use of corticosteroids more than 5 mg prednisolone per day.
	Atypical fracture or osteonecrosis of the jaw.
	Expected follow-op period less than 3 years (e.g. due to uncontrolled malignancy).
	Expected to require special postoperative surveillance due to increased surgical risk (e.g. for cardiac, psychiatric condition).

For peer review only

Randomization procedure and blinding

When found eligible, patients will be randomized to either zoledronic acid or placebo through block randomization by the study nurse on the day of surgery. Block randomization will be used to label infusion bags for drug delivery. The type of implant (hip or knee, cemented or not) will be a stratification factor in the randomization to ensure balance among these factors. All staff involved in patient care are blinded to treatment. The nurse in the postoperative care unit who is responsible for the preparation of the study drug according to the randomization list will not be blinded. However, because this person is not otherwise involved in the study, concealment of treatment allocation is not jeopardized. The content of the infusion bag will be administered to the patient on the day after surgery by a blinded nurse in the surgical ward. The randomization list will be available for unblinding in emergency situations 24 h a day at *Apoteksbolaget AB* at Linköping University Hospital.

Intervention

Patients will be randomized to receive a single postoperative infusion of zoledronic acid 4 mg/5 ml(17) or placebo (5ml saline) on the day after surgery.

Study Outcomes

Rationale for the outcome measures

In previous epidemiological studies of prosthetic loosening the endpoint has been revision surgery. We will report this parameter continuously and we will use the Swedish hip and knee arthroplasty registries to capture reoperations performed outside our uptake area. Since the overall revision rate for aseptic loosening in Sweden is around 2-3% during a 10-year period, this endpoint would demand not only a very large study sample but also a long-term follow-up to get sufficient power. Also, some patients with loosening do not undergo revision surgery. They might be too old or fragile for these demanding operations. Other patients only have modest symptoms and might refrain from a demanding reoperation. Therefore, another primary outcome must be considered for reasons of feasibility. A previous study with the same treatment protocol of zoledronic acid as ours reported not only less implant migration in the zoledronic acid group but also a statistically significant improvement on the Harris Hip Score(17) two and three years postoperatively, despite small numbers (n=50). This study comprised only uncemented prostheses in younger patients with osteonecrosis of the femoral head. Based on these findings, because of the clinical importance and the predictive value on future revision surgery, *(25-28)* we have chosen to use patient reported scores as our primary outcome: Hip dysfunction and Osteoarthritis Outcome Score (HOOS), Swedish version LK 2.0(29) and the Knee Injury and Osteoarthritis Outcome Score (KOOS), Swedish version LK1.0.(30) Both instruments were meticulously designed with items generated in an iterative process including input from stakeholder groups comprised of patients, orthopaedists, and physical therapists. Both instruments have undergone extensive psychometric testing(31) and are recommended for evaluation of TJR by the International Consortium for Health Outcomes Measurement. These measures are free to use and have previously been shown to be highly reliable, with excellent internal consistency (Cronbach's alpha coefficient of 0.82-0.98) in samples of people undergoing THR and TKR.(32, 33)

Our primary outcome measure will be between-group differences in KOOS/HOOS from baseline until the 3-year follow-up. Based on our literature review at the time of study design and confirmed later by the *Outcomes Measures in Rheumatology*, *Workgroup Total Joint Replacement*,(34) the subscale *pain* in HOOS/KOOS will be analysed as the primary endpoint in the confirmative analysis.

For peer review only - http://bmjopen.bmj.com/site/about/guidelines.xhtml

Secondary endpoints are included for supportive evidence. As secondary endpoints, we will analyse between-group differences in the remaining subscales of the KOOS/HOOS from baseline to 3 years, all subscales of the KOOS/HOOS and the RAND/SF36 (Swedish version from 2013-05-21, using the 4 week recall period)(35, 36) at 1, 3 and 6 years and signs of radiographic loosening at 3 and 6 years (Table 2). The RAND/SF36 will be analysed using physical and mental component scores.

For peer review only

Table 2. Schedule of assessments and events.

**Visit**	1	2	3	4	5	6	7	8
	Screening	Surgery	Treatment	Discharge	Follow-up	Follow-up	Follow-up	Follow-up
**Time**	Day -28 to - 2	Day 1	Day 2	Day 3-5	6 weeks	1 year ±1 month	3 year ±1 month	6 year ±1 month
**Assessment /event**								
Informed Consent*[1]	X							
Demography Medical history	X							
Physical Examination	X							
Height and Weight	X							
Assessment of inclusion and exclusion criteria	X	X						
Blood sampling	X		X					
Start of continuous daily Calcium*[2]			X					
Surgery		X						
Randomization*[3]		X	X					
Administration of IMP/Placebo*[4]			X					
HOOS/KOOS RAND/SF-36 questionnaire	X					X	X	X
X-ray		X					X	X
Concomitant Medication	X	X						
Routine follow-up (via phone)						X		
AE Assessment*[5]			X	X	X	X	X	X

IMP = investigational medical product, HOOS = Hip dysfunction and Osteoarthritis Outcome Score, KOOS = Knee injury and Osteoarthritis Outcome Score, SF-36 = 36 item Short Form, X-ray = Plain radiography, AE = Adverse Event, SAE = Severe Adverse Event.

*[1] The Informed Consent Form must be signed before any study related procedure

*[2] Vitamin D and calcium will be given daily in standard dosage from day 2 for 1 month.

*[3] Randomization has to be performed as close as possible prior to the first IMP infusion

*[4] Administration of IMP/Placebo will be given the day after surgery.

*[5] AE/SAE will be collected via a questionnaire at visit 5 and personal interview at 4. At visit 6, 7 and 8 only SAE will be collected via a questionnaire. Reminders will be given by phone.

For peer review only

Statistical analysis

Power

Both HOOS and KOOS ranges from 0 to 100. The minimal important change is often reported to be 8-10 points but this estimate is dependent on contextual factors such as patient age, intervention and time to follow-up, and according to the developers of KOOS (www.koos.nu) no generic value of the minimal important change is available for KOOS, or any other patient reported outcome measure. In a large sample a small statistically significant difference is still indicative of an important treatment effect, even if the difference is smaller than the minimal clinically important difference. At the time of the study design, average reported KOOS values for 3 years after TKR were not available, and sample size calculations therefore were based on average values 2 years after TKR: 84, (SD 14). Lacking official consensus on a recommended clinically relevant difference the research team decided upon a 3-point difference on the HOOS/KOOS scale after 3 years. These values would with Student's t-test and a two-sided significance level of 5 % yield 90 % power when 450 patients are included in each arm. To compensate for a 10% withdrawal, a further 50 patients would be needed, leading to a total of 1000 patients to include in a superiority trial.

Statistical analysis plan

Statistical analysis of the primary endpoint will be carried out using a mixed model repeated measurements ANOVA of the changes in KOOS/HOOS from baseline until 3-years follow-up with covariate adjustment for baseline values of HOOS/KOOS, and with implant type (hip, knee and cemented, uncemented) and age (continuous) as further covariates. As supportive endpoints, we will analyse HOOS/KOOS subscales at 6-years follow-up, signs of radiographic loosening at 3 and 6 years and SF/RAND36 at 1, 3 and 6 years. we will also perform subgroup analysis of men and women and implant types.

To reduce the risk of bias during interpretation, blinded results from the analyses (with study groups labelled as group A and group B) will be presented to all the authors, who will agree in writing on two alternative interpretations.(37) Thereafter, the data manager will break the randomization code.

As part of an exploratory analysis and to be able to define the clinical impact of our results we will perform a responder analysis comparing the proportion of patients who achieve a substantial clinical improvement on the subscale *pain* in HOOS/KOOS between the treatment and the control group.(38) HOOS and KOOS values depend on age, BMI and sex, which will be considered as cofactors in the analysis.

No interim analysis will be performed.

Safety

Concomitant drug treatment

After the infusion, oral supplements of vitamin D and calcium will be given once daily to both groups for the first postoperative month to prevent bisphosphonate-induced hypocalcaemia.

Zoledronic acid

Repeated infusions of zoledronic acid has been associated with a slight increase in atrial fibrillation in the highest age groups(39) and because of this we have set an upper age limit for inclusion to 80 years. Bisphosphonate use is strongly associated with osteonecrosis of the jaw. This is however a very rare condition and only associated with multiple dosing over

For peer review only

time.(40) 10% of patients treated with zoledronic acid have reported Acute-Phase Reactions (41) which can lead to a prolonged hospital stay. Even though zoledronic acid has not been reported to cause hypocalcaemia in osteoporosis treatment,(42) normal preoperative calcium and vitamin D levels are required for inclusion for safety reasons and patients will get oral supplements for the first postoperative month. Zoledronic acid can affect kidney function and the manufacturer recommends against its use in patients with creatinine clearance <35 ml/min.(43) Bisphosphonates are used in large scale to treat patients with osteoporosis, and its safety is extensively documented.(40) Furthermore, in this study only one dose of 4mg(17) is given, compared to the repeated dosing of 5mg in osteoporosis treatment.

Adverse events

Patients will be followed-up for 6 weeks after the infusion for adverse events (AE). The physiotherapist will record AE´s at the 6-week follow-up (Table 2). An AE is defined in the International Conference on Harmonization (ICH) Guidelines for Good Clinical Practice as "any untoward medial occurrence in a patient or clinical investigation subject administered a pharmaceutical product and does not necessarily have a causal relationship with this treatment". The occurrence of atypical femoral fractures and osteonecrosis of the jaw will be recorded throughout the whole study period. Serious adverse event (SAE) is defined as an AE that is fatal, life threatening, requires in-patient hospitalization or prolonged hospitalization, results in persistent or significant disability/incapacity or other significant medical hazards. Adverse Drug Reaction (ADR) is defined as all untoward and unintended response to a medical product related to any dose administered and will also be recorded during the first 6 weeks. The occurrence of AE and SAE will be presented descriptively. For any harm caused through study participation, all patients are covered by the national Swedish patient insurance, *Landstingens Ömsesidiga Försäkringsbolag.*

ETHICS AND DISSEMINATION

Ethics

The study will be conducted in agreement with the Helsinki declaration and ICH guidelines will be adhered to. The study will be monitored by *Forum Östergötland,* which is part of the national organisation for Clinical Studies in Sweden, Forum Sydost. All completed questionnaires will be kept secured from unauthorized access within the research nurses´ facility. Data for the purpose of statistical analyses will be collected in digitized files. Other data will be stored in the patients´ ordinary medical chart. The study has been approved by the Regional Ethical Review Board in Linköping (DNR 2015/286-31).

Dissemination

The results of this study will be submitted for publication in an international peer reviewed scientific paper regardless of whether the results are positive, negative or inconclusive regarding the hypothesis of the study.

Patient and Public Involvement

No patient organisation or patient representatives were involved in the design of the study. The results of our study will be disseminated to patient organizations and the public through the Swedish Orthopaedic Association and the Swedish National Joint Arthroplasty Register.

For peer review only

DISCUSSION

Strengths and limitations

Strengths

The main strength of this study is its size and design. To our knowledge this is the largest RCT designed to elucidate if bisphosphonates can improve outcome in primary THA and TKA. If we can demonstrate a significant increase in patient satisfaction after bisphosphonate administration, it could revolutionize the perioperative care of patients undergoing TJR.

Limitations

The main limitation of this study can be considered its primary patient-reported outcome measure. A hard endpoint as a prospectively collected rate of revision would be preferable, however not feasible in this research question. Also, not all patients with loosening of their implants undergo revision surgery. Some are too old and fragile, and others have moderate symptoms and might decide to abstain from surgery. The use of a patient-reported primary endpoint will increase the relevance of the findings to patients. Improved patient reported outcomes after TJR might in fact be more important for the majority of the patients undergoing TJR compared to prosthetic loosening assessed on radiographs, which is a secondary outcome. If we fail to demonstrate a significant increase in patient reported outcomes, we might be able to show a decrease in radiographic signs of early loosening, which strongly correlates with late aseptic loosening.(13, 14) Also, dual-energy x-ray absorptiometry will not be performed.

Authors affiliation

1 Department of Orthopaedic Surgery, Capio Specialistvård Motala, Motala, Sweden

2 Department of Orthopaedics and Experimental and Clinical Medicine, Faculty of Health Science, Linköping University, Linköping, Sweden

3 Department of Clinical Sciences Lund, Orthopaedics, Lund University, Lund, Sweden

4 Research Unit for Musculoskeletal Function and Physiotherapy, Institute of Sports Science and Clinical Biomechanics, University of Southern Denmark, Odense, Denmark

Author contributions

JB: Writing of manuscript, study design
HL: Writing of manuscript, study design
JR: Revision of manuscript, study design
ER: Revision of manuscript, study design
PA: Study design, preparation of manuscript
JS: Writing and revision of manuscript, study design

Funding statement

This academic drug trial is run without any support from the industry. Financial support was received from the Swedish Research Council, grant number 2014-07284, and ALF-grants from Region Östergötland, Sweden.

For peer review only

Data statement

The dataset will be made available in a data repository.

Competing interests

None stated. Data collection, management, analysis, and interpretation of data; writing of the report; and the decision to submit the report for publication are the sole authority of the study sponsor.

Acknowledgements

We are grateful for the research-friendly leadership of the healthcare providers Capio Specialistvård Motala and Aleris Specialistvård Motala. We also thank all personnel at Motala hospital for their extra effort to make this large research project possible, in specific our research nurses Anneli Kvarnestedt and Carmen Henriksson.

REFERENCES

1. Learmonth ID, Young C, Rorabeck C. The operation of the century: total hip replacement. *Lancet* 2007;370:1508-19.
2. Evans JT, Walker RW, Evans JP, et al. How long does a knee replacement last? A systematic review and meta-analysis of case series and national registry reports with more than 15 years of follow-up. *The Lancet* 2019;393:655-63.
3. Beswick AD, Wylde V, Gooberman-Hill R, et al. What proportion of patients report long-term pain after total hip or knee replacement for osteoarthritis? A systematic review of prospective studies in unselected patients. *BMJ Open* 2012;2:e000435.
4. Kärrholm J MM, Odin M, Vinblad J, Rogmark C, Rolfson O. The Swedish Hip Arthroplasty Register, Annual Report 2017. 2018.
5. Robertsson O LL, Sundberg M, W-Dahl A,. The Swedish Knee Arthroplasty Register, Annual Report 2019. 2019.
6. Vanhegan IS, Malik AK, Jayakumar P, et al. A financial analysis of revision hip arthroplasty: the economic burden in relation to the national tariff. *The Journal of bone and joint surgery British volume* 2012;94:619-23.
7. Lenguerrand E, Whitehouse MR, Wylde V, et al. Pain and Function Recovery Trajectories following Revision Hip Arthroplasty: Short-Term Changes and Comparison with Primary Hip Arthroplasty in the ADAPT Cohort Study. *PloS one* 2016;11:e0164839.
8. Ong KL, Lau E, Suggs J, et al. Risk of subsequent revision after primary and revision total joint arthroplasty. *Clin Orthop Relat Res* 2010;468:3070-6.
9. Bernhardsson M, Sandberg O, Aspenberg P. Experimental models for cancellous bone healing in the rat. *Acta Orthop* 2015;86:745-50.
10. Aspenberg P, Van der Vis H. Migration, particles, and fluid pressure. A discussion of causes of prosthetic loosening. *Clinical orthopaedics and related research* 1998:75-80.
11. Schindeler A, McDonald MM, Bokko P, et al. Bone remodeling during fracture repair: The cellular picture. *Semin Cell Dev Biol* 2008;19:459-66.
12. Agholme F, Aspenberg P. Experimental results of combining bisphosphonates with allograft in a rat model. *J Bone Joint Surg Br* 2009;91:670-5.
13. Pijls BG, Nieuwenhuijse MJ, Fiocco M, et al. Early proximal migration of cups is associated with late revision in THA: a systematic review and meta-analysis of 26 RSA studies and 49 survivalstudies. *Acta Orthop* 2012;83:583-91.
14. Pijls BG, Valstar ER, Nouta KA, et al. Early migration of tibial components is associated with late revision: a systematic review and meta-analysis of 21,000 knee arthroplasties. *Acta Orthop* 2012;83:614-24.
15. Yu NY, Schindeler A, Tagil M, et al. Use of BMPs and bisphosphonates in improving bone fracture healing. *Front Biosci (Elite Ed)* 2012;4:2647-53.

For peer review only

16. Hilding M, Aspenberg P. Local peroperative treatment with a bisphosphonate improves the fixation of total knee prostheses: a randomized, double-blind radiostereometric study of 50 patients. *Acta Orthop* 2007;78:795-9.
17. Friedl G, Radl R, Stihsen C, et al. The effect of a single infusion of zoledronic acid on early implant migration in total hip arthroplasty. A randomized, double-blind, controlled trial. *The Journal of bone and joint surgery American volume* 2009;91:274-81.
18. Schilcher J, Palm L, Ivarsson I, et al. Local bisphosphonate reduces migration and formation of radiolucent lines adjacent to cemented acetabular components. *Bone Joint J* 2017;99-b:317-24.
19. Abtahi J, Tengvall P, Aspenberg P. A bisphosphonate-coating improves the fixation of metal implants in human bone. A randomized trial of dental implants. *Bone* 2012;50:1148-51.
20. Thillemann TM, Pedersen AB, Mehnert F, et al. Postoperative use of bisphosphonates and risk of revision after primary total hip arthroplasty: a nationwide population-based study. *Bone* 2010;46:946-51.
21. Prieto-Alhambra D, Javaid MK, Judge A, et al. Association between bisphosphonate use and implant survival after primary total arthroplasty of the knee or hip: population based retrospective cohort study. *BMJ* 2011;343:d7222.
22. Prieto-Alhambra D, Lalmohamed A, Abrahamsen B, et al. Oral bisphosphonate use and total knee/hip implant survival: validation of results in an external population-based cohort. *Arthritis & rheumatology* 2014;66:3233-40.
23. Khatod M, Inacio MC, Dell RM, et al. Association of Bisphosphonate Use and Risk of Revision After THA: Outcomes From a US Total Joint Replacement Registry. *Clin Orthop Relat Res* 2015;473:3412-20.
24. Teng S, Yi C, Krettek C, et al. Bisphosphonate Use and Risk of Implant Revision after Total Hip/Knee Arthroplasty: A Meta-Analysis of Observational Studies. *PloS one* 2015;10:e0139927.
25. Rothwell AG, Hooper GJ, Hobbs A, et al. An analysis of the Oxford hip and knee scores and their relationship to early joint revision in the New Zealand Joint Registry. *The Journal of bone and joint surgery British volume* 2010;92:413-8.
26. Rolfson O, Bohm E, Franklin P, et al. Patient-reported outcome measures in arthroplasty registries Report of the Patient-Reported Outcome Measures Working Group of the International Society of Arthroplasty Registries Part II. Recommendations for selection, administration, and analysis. *Acta Orthop* 2016;87 Suppl 1:9-23.
27. Devane P, Horne G, Gehling DJ. Oxford hip scores at 6 months and 5 years are associated with total hip revision within the subsequent 2 years. *Clinical orthopaedics and related research* 2013;471:3870-4.
28. Singh JA, Schleck C, Harmsen S, et al. Clinically important improvement thresholds for Harris Hip Score and its ability to predict revision risk after primary total hip arthroplasty. *BMC Musculoskelet Disord* 2016;17:256.
29. Nilsdotter AK, Lohmander LS, Klassbo M, et al. Hip disability and osteoarthritis outcome score (HOOS)--validity and responsiveness in total hip replacement. *BMC Musculoskelet Disord* 2003;4:10.
30. Roos EM, Toksvig-Larsen S. Knee injury and Osteoarthritis Outcome Score (KOOS) - validation and comparison to the WOMAC in total knee replacement. *Health Qual Life Outcomes* 2003;1:17.
31. Losina E, Ranstam J, Collins JE, et al. OARSI Clinical Trials Recommendations: Key analytic considerations in design, analysis, and reporting of randomized controlled trials in osteoarthritis. *Osteoarthritis and cartilage* 2015;23:677-85.
32. Nilsdotter A, Bremander A. Measures of hip function and symptoms: Harris Hip Score (HHS), Hip Disability and Osteoarthritis Outcome Score (HOOS), Oxford Hip Score (OHS), Lequesne Index of Severity for Osteoarthritis of the Hip (LISOH), and American Academy of Orthopedic Surgeons (AAOS) Hip and Knee Questionnaire. *Arthritis Care Res (Hoboken)* 2011;63 Suppl 11:S200-7.
33. Collins NJ, Roos EM. Patient-reported outcomes for total hip and knee arthroplasty: commonly used instruments and attributes of a "good" measure. *Clinics in geriatric medicine* 2012;28:367-94.

For peer review only

34. Singh JA, Dowsey MM, Dohm M, et al. Achieving Consensus on Total Joint Replacement Trial Outcome Reporting Using the OMERACT Filter: Endorsement of the Final Core Domain Set for Total Hip and Total Knee Replacement Trials for Endstage Arthritis. *The Journal of rheumatology* 2017;44:1723-6.
35. Hays RD, Sherbourne CD, Mazel RM. The RAND 36-Item Health Survey 1.0. *Health Econ* 1993;2:217-27.
36. Ware JE, Jr., Gandek B. Overview of the SF-36 Health Survey and the International Quality of Life Assessment (IQOLA) Project. *J Clin Epidemiol* 1998;51:903-12.
37. Jarvinen TL, Sihvonen R, Bhandari M, et al. Blinded interpretation of study results can feasibly and effectively diminish interpretation bias. *J Clin Epidemiol* 2014;67:769-72.
38. Lyman S, Lee YY, McLawhorn AS, et al. What Are the Minimal and Substantial Improvements in the HOOS and KOOS and JR Versions After Total Joint Replacement? *Clinical orthopaedics and related research* 2018;476:2432-41.
39. Kim DH, Rogers JR, Fulchino LA, et al. Bisphosphonates and risk of cardiovascular events: a meta-analysis. *PLoS One* 2015;10:e0122646.
40. Reyes C, Hitz M, Prieto-Alhambra D, et al. Risks and Benefits of Bisphosphonate Therapies. *J Cell Biochem* 2016;117:20-8.
41. Kotian P, Boloor A, Sreenivasan S. Study of Adverse Effect Profile of Parenteral Zoledronic Acid in Female Patients with Osteoporosis. *J Clin Diagn Res* 2016;10:OC04-6.
42. Kreutle V, Blum C, Meier C, et al. Bisphosphonate induced hypocalcaemia - report of six cases and review of the literature. *Swiss Med Wkly* 2014;144:w13979.
43. McClung M, Harris ST, Miller PD, et al. Bisphosphonate therapy for osteoporosis: benefits, risks, and drug holiday. *Am J Med* 2013;126:13-20.

For peer review only

Patientens namn: ……………………………

Studienummer: ……………………………

Patientinformation

Du har artros i en höft- eller knäled. Tillsammans med din behandlande läkare har ni kommit fram till att du behöver opereras med en protes.
Denna patientinformation är en förfrågan om att delta i en forskningsstudie.

Zoledronat är ett läkemedel registrerat för behandling av benskörhet. Det verkar genom att minska den naturliga nedbrytningen av skelettet som sker med åldern. Läkemedlet verkar också kunna förbättra fastläkning av ledproteser.

Syftet med denna studie är att ta reda på om Zoledronat kan förbättra resultat efter protesoperationer i höft- eller knäled. Vi planerar att inkludera 1000 patienter som behöver en höft- eller knäprotes på grund artros. Hälften av patienterna får behandling med Zoledronat dagen efter operation och den andra hälften får endast koksalt (overksamt läkemedel). Behandlingen sker genom en långsam injektion (infusion). I övrigt påverkas inte ditt omhändertagande före eller efter operationen, utöver att vi kommer att kontrollera den opererade leden med röntgen efter 3 år och 6 år. Du kommer också att få 2 formulär hemskickat till dig vid 1, 3 och 6 år efter operation. Med dessa formulär vill vi på ett standardiserat sätt få reda på hur du upplever resultatet av din operation.

Behandling med Zoledronat kan ge biverkningar. Vanliga biverkningar efter den första infusionen är feber och huvudvärk (influensaliknande). De flesta av dessa biverkningar uppträder inom de tre första dagarna efter behandlingen och upphör inom 3 dagar efter att de började. Genom den smärtlindring du får på grund av operationen kommer du sannolikt inte märka dessa biverkningar alls. Sällsynta och allvarliga biverkningar vid behandling med Zoledronat är frakturer i lårbenet och nedbrytning av käkbenet. Dessa förekommer dock endast vid upprepade behandlingar och i kombination med allvarliga grundsjukdomar. I denna studie ges zoledronat som en engångsdos, dagen efter din operation.

Om du vill delta, kommer du att lottas till antingen behandling med placebo (koksalt) eller Zoledronat. Varken Du eller din opererande läkare vet vilken behandling Du får.

Ditt deltagande är helt frivilligt. Om du väljer att inte delta kommer du att omhändertas på sedvanligt sätt. Du kan när som helst under studien avbryta ditt deltagande utan att det påverkar din behandling.

Version 1.5 2019-04-01

For peer review only - http://bmjopen.bmj.com/site/about/guidelines.xhtml

Om Du beslutar dig för att avbryta studien, kommer din doktor att be dig om tillåtelse att samla in information från dina journalhandlingar. Studien är godkänd av Etikprövningsnämnd och tillstånd har lämnats av Läkemedelsverket. Du är som patient försäkrad genom Läkemedelsförsäkringen och Patientskadelagen.

Behandling av personuppgifter

Under studien kommer ansvarig läkare att samla in uppgifter om födelsedatum, kön, hälsodata (såsom t.ex. tidigare sjukdomar och läkemedelsanvändning) samt resultat av undersökningar i studien.

Uppgifter insamlas i studien utan ditt namn eller personnummer men med en kod. Endast ansvarig läkare har tillgång till din ”nyckel”, med vilken det går att koppla uppgifterna till dig. Capio Specialistvård i Motala AB är personuppgifts-ansvariga för behandling av personuppgifter.

Ändamålen med detta register är forskning och utveckling av läkemedel som beskrivits i denna information samt godkännande/registrering av kommande produkter och säkerhetsuppföljning, därmed är allmänt intresse den rättsliga grunden för hantering av personuppgifter. Resultat kan också komma att publiceras i någon medicinsk tidskrift utan att din identitet uppges.

Uppgifterna hanteras enligt Dataskyddsförordningen, GDPR (EU 2016/679) och du har rätt att få veta vilka uppgifter som samlas in om dig, begära rättelse vid eventuella felaktigheter eller begära begränsning/ borttagning av uppgifter.

Ansvarig läkare:
Håkan Ledin, Mobil: 072-204 56 35; hakan.ledin@regionostergotland.se
Bengt Horn, Mobil: 070-158 63 54 Bengt.Horn.af.Aminne@regionostergotland.se

Skriftligt Samtycke

Jag har tagit del av informationen och accepterar att deltaga i studien. Jag har också informerats om och samtyckt till att en oberoende granskare (monitor) och läkemedelsmyndighet vid behov får jämföra de i studien rapporterade uppgifterna med de som finns i min patientjournal. Detta får ske under förbehåll att den information som då blir tillgänglig inte förs vidare.

____

Underskrift Patient Namnförtydligande Datum

____

Underskrift Läkare Namnförtydligande Datum

Version 1.5 2019-04-01

For peer review only - http://bmjopen.bmj.com/site/about/guidelines.xhtml

Reporting checklist for protocol of a clinical trial.

Based on the SPIRIT guidelines.

Instructions to authors

Complete this checklist by entering the page numbers from your manuscript where readers will find each of the items listed below.

Your article may not currently address all the items on the checklist. Please modify your text to include the missing information. If you are certain that an item does not apply, please write "n/a" and provide a short explanation.

Upload your completed checklist as an extra file when you submit to a journal.

In your methods section, say that you used the SPIRITreporting guidelines, and cite them as:

Chan A-W, Tetzlaff JM, Altman DG, Laupacis A, Gøtzsche PC, Krleža-Jerić K, Hróbjartsson A, Mann H, Dickersin K, Berlin J, Doré C, Parulekar W, Summerskill W, Groves T, Schulz K, Sox H, Rockhold FW, Rennie D, Moher D. SPIRIT 2013 Statement: Defining standard protocol items for clinical trials. Ann Intern Med. 2013;158(3):200-207

For peer review only

		Reporting Item	Page Number
**Administrative information**			
Title	#1	Descriptive title identifying the study design, population, interventions, and, if applicable, trial acronym	1
Trial registration	#2a	Trial identifier and registry name. If not yet registered, name of intended registry	2
Trial registration: data set	#2b	All items from the World Health Organization Trial Registration Data Set	2
Protocol version	#3	Date and version identifier	2
Funding	#4	Sources and types of financial, material, and other support	10
Roles and responsibilities: contributorship	#5a	Names, affiliations, and roles of protocol contributors	9

Roles and responsibilities: sponsor contact information	#5b	Name and contact information for the trial sponsor	1
Roles and responsibilities: sponsor and funder	#5c	Role of study sponsor and funders, if any, in study design; collection, management, analysis, and interpretation of data; writing of the report; and the decision to submit the report for publication, including whether they will have ultimate authority over any of these activities	9
Roles and responsibilities: committees	#5d	Composition, roles, and responsibilities of the coordinating centre, steering committee, endpoint adjudication committee, data management team, and other individuals or groups overseeing the trial, if applicable (see Item 21a for data monitoring committee)<br><br>**No committees involved**	n/a
**Introduction**			
Background and rationale	#6a	Description of research question and justification for undertaking the trial, including summary of relevant studies (published and unpublished) examining benefits and harms for each intervention	3
Background and rationale: choice of comparators	#6b	Explanation for choice of comparators	5
Objectives	#7	Specific objectives or hypotheses	3
Trial design	#8	Description of trial design including type of trial (eg, parallel group, crossover, factorial, single group), allocation ratio, and framework (eg, superiority, equivalence, non-inferiority, exploratory)	3
**Methods: Participants, interventions, and outcomes**			
Study setting	#9	Description of study settings (eg, community clinic, academic hospital) and list of countries where data will be collected. Reference to where list of study sites can be obtained	3

For peer review only

Eligibility criteria	#10	Inclusion and exclusion criteria for participants. If applicable, eligibility criteria for study centres and individuals who will perform the interventions (eg, surgeons, psychotherapists)	4
Interventions: description	#11a	Interventions for each group with sufficient detail to allow replication, including how and when they will be administered	4
Interventions: modifications	#11b	Criteria for discontinuing or modifying allocated interventions for a given trial participant (eg, drug dose change in response to harms, participant request, or improving / worsening disease) **Only one dose is given**	n/a
Interventions: adherance	#11c	Strategies to improve adherence to intervention protocols, and any procedures for monitoring adherence (eg, drug tablet return; laboratory tests)	6
Interventions: concomitant care	#11d	Relevant concomitant care and interventions that are permitted or prohibited during the trial	4
Outcomes	#12	Primary, secondary, and other outcomes, including the specific measurement variable (eg, systolic blood pressure), analysis metric (eg, change from baseline, final value, time to event), method of aggregation (eg, median, proportion), and time point for each outcome. Explanation of the clinical relevance of chosen efficacy and harm outcomes is strongly recommended	5
Participant timeline	#13	Time schedule of enrolment, interventions (including any run-ins and washouts), assessments, and visits for participants. A schematic diagram is highly recommended (see Figure)	6
Sample size	#14	Estimated number of participants needed to achieve study objectives and how it was determined, including clinical and statistical assumptions supporting any sample size calculations	7
Recruitment	#15	Strategies for achieving adequate participant enrolment to reach target sample size	4
**Methods: Assignment of interventions (for controlled trials)**			
Allocation: sequence generation	#16a	Method of generating the allocation sequence (eg, computer-generated random numbers), and list of any factors for	4

For peer review only

		stratification. To reduce predictability of a random sequence, details of any planned restriction (eg, blocking) should be provided in a separate document that is unavailable to those who enrol participants or assign interventions	
Allocation concealment mechanism	#16b	Mechanism of implementing the allocation sequence (eg, central telephone; sequentially numbered, opaque, sealed envelopes), describing any steps to conceal the sequence until interventions are assigned	4
Allocation: implementation	#16c	Who will generate the allocation sequence, who will enrol participants, and who will assign participants to interventions	4
Blinding (masking)	#17a	Who will be blinded after assignment to interventions (eg, trial participants, care providers, outcome assessors, data analysts), and how	4
Blinding (masking): emergency unblinding	#17b	If blinded, circumstances under which unblinding is permissible, and procedure for revealing a participant's allocated intervention during the trial	4
**Methods: Data collection, management, and analysis**			
Data collection plan	#18a	Plans for assessment and collection of outcome, baseline, and other trial data, including any related processes to promote data quality (eg, duplicate measurements, training of assessors) and a description of study instruments (eg, questionnaires, laboratory tests) along with their reliability and validity, if known. Reference to where data collection forms can be found, if not in the protocol	6
Data collection plan: retention	#18b	Plans to promote participant retention and complete follow-up, including list of any outcome data to be collected for participants who discontinue or deviate from intervention protocols	6
Data management	#19	Plans for data entry, coding, security, and storage, including any related processes to promote data quality (eg, double data entry; range checks for data values). Reference to where details of data management procedures can be found, if not in the protocol	9

For peer review only

Statistics: outcomes	#20a	Statistical methods for analysing primary and secondary outcomes. Reference to where other details of the statistical analysis plan can be found, if not in the protocol	5
Statistics: additional analyses	#20b	Methods for any additional analyses (eg, subgroup and adjusted analyses)	5
Statistics: analysis population and missing data	#20c	Definition of analysis population relating to protocol non-adherence (eg, as randomised analysis), and any statistical methods to handle missing data (eg, multiple imputation)	5
**Methods: Monitoring**			
Data monitoring: formal committee	#21a	Composition of data monitoring committee (DMC); summary of its role and reporting structure; statement of whether it is independent from the sponsor and competing interests; and reference to where further details about its charter can be found, if not in the protocol. Alternatively, an explanation of why a DMC is not needed	8
Data monitoring: interim analysis	#21b	Description of any interim analyses and stopping guidelines, including who will have access to these interim results and make the final decision to terminate the trial	7
Harms	#22	Plans for collecting, assessing, reporting, and managing solicited and spontaneously reported adverse events and other unintended effects of trial interventions or trial conduct	8
Auditing	#23	Frequency and procedures for auditing trial conduct, if any, and whether the process will be independent from investigators and the sponsor	8
**Ethics and dissemination**			
Research ethics approval	#24	Plans for seeking research ethics committee / institutional review board (REC / IRB) approval	8
Protocol amendments	#25	Plans for communicating important protocol modifications (eg, changes to eligibility criteria, outcomes, analyses) to relevant parties (eg, investigators, REC / IRBs, trial participants, trial 8registries, journals, regulators)	8

For peer review only

Consent or assent	#26a	Who will obtain informed consent or assent from potential trial participants or authorised surrogates, and how (see Item 32)	3
Consent or assent: ancillary studies	#26b	Additional consent provisions for collection and use of participant data and biological specimens in ancillary studies, if applicable	
Confidentiality	#27	How personal information about potential and enrolled participants will be collected, shared, and maintained in order to protect confidentiality before, during, and after the trial	8
Declaration of interests	#28	Financial and other competing interests for principal investigators for the overall trial and each study site	9
Data access	#29	Statement of who will have access to the final trial dataset, and disclosure of contractual agreements that limit such access for investigators	9
Ancillary and post trial care	#30	Provisions, if any, for ancillary and post-trial care, and for compensation to those who suffer harm from trial participation	9
Dissemination policy: trial results	#31a	Plans for investigators and sponsor to communicate trial results to participants, healthcare professionals, the public, and other relevant groups (eg, via publication, reporting in results databases, or other data sharing arrangements), including any publication restrictions	9
Dissemination policy: authorship	#31b	Authorship eligibility guidelines and any intended use of professional writers	9
Dissemination policy: reproducible research	#31c	Plans, if any, for granting public access to the full protocol, participant-level dataset, and statistical code	9
**Appendices**			
Informed consent materials	#32	Model consent form and other related documentation given to participants and authorised surrogates	4
Biological specimens	#33	Plans for collection, laboratory evaluation, and storage of biological specimens for genetic or molecular analysis in the current trial and for future use in ancillary studies, if applicable<br><br>**No samples are stored for study purposes**	n/a

For peer review only

None The SPIRIT checklist is distributed under the terms of the Creative Commons Attribution License CC-BY-ND 3.0. This checklist can be completed online using https://www.goodreports.org/, a tool made by the EQUATOR Network in collaboration with Penelope.ai

For peer review only

BMJ Open

A single postoperative infusion of zoledronic acid to improve patient reported outcome after hip or knee replacement: study protocol for a randomized, controlled, double-blinded clinical trial.

Journal:	*BMJ Open*
Manuscript ID	bmjopen-2020-040985.R2
Article Type:	Protocol
Date Submitted by the Author:	23-Aug-2020
Complete List of Authors:	Brandt, Jonathan; Capio Specialistvård Motala, Department of Orthopaedic Surgery<br>Ledin, Håkan; Capio Specialistvård Motala, Department of Orthopaedic Surgery<br>Ranstam, Jonas; Lunds Universitet, Clinical sciences<br>Roos, Ewa; Syddansk Universitet Det Sundhedsvidenskabelige Fakultet, Sports Science and Clinical Biomechanics<br>Aspenberg, Per; Linköping University Hospital, Department of Orthopaedics in Linköping, and Department of Biomedical and Clinical Sciences, Linköping University<br>Schilcher, Jörg; Linköping University Hospital, Department of Orthopaedics and Department of Biomedical and Clinical Sciences, Faculty of Health Science
**Primary Subject Heading**:	Surgery
Secondary Subject Heading:	Complementary medicine
Keywords:	Adult orthopaedics < ORTHOPAEDIC & TRAUMA SURGERY, Hip < ORTHOPAEDIC & TRAUMA SURGERY, Knee < ORTHOPAEDIC & TRAUMA SURGERY

SCHOLARONE™
Manuscripts

I, the Submitting Author has the right to grant and does grant on behalf of all authors of the Work (as defined in the below author licence), an exclusive licence and/or a non-exclusive licence for contributions from authors who are: i) UK Crown employees; ii) where BMJ has agreed a CC-BY licence shall apply, and/or iii) in accordance with the terms applicable for US Federal Government officers or employees acting as part of their official duties; on a worldwide, perpetual, irrevocable, royalty-free basis to BMJ Publishing Group Ltd ("BMJ") its licensees and where the relevant Journal is co-owned by BMJ to the co-owners of the Journal, to publish the Work in this journal and any other BMJ products and to exploit all rights, as set out in our licence.

The Submitting Author accepts and understands that any supply made under these terms is made by BMJ to the Submitting Author unless you are acting as an employee on behalf of your employer or a postgraduate student of an affiliated institution which is paying any applicable article publishing charge ("APC") for Open Access articles. Where the Submitting Author wishes to make the Work available on an Open Access basis (and intends to pay the relevant APC), the terms of reuse of such Open Access shall be governed by a Creative Commons licence – details of these licences and which Creative Commons licence will apply to this Work are set out in our licence referred to above.

Other than as permitted in any relevant BMJ Author's Self Archiving Policies, I confirm this Work has not been accepted for publication elsewhere, is not being considered for publication elsewhere and does not duplicate material already published. I confirm all authors consent to publication of this Work and authorise the granting of this licence.

For peer review only

A single postoperative infusion of zoledronic acid to improve patient reported outcome after hip or knee replacement: study protocol for a randomized, controlled, double-blinded clinical trial.

For peer review only

Jonathan Brandt M.D.[1,2], Håkan Ledin M.D. [1,2], Jonas Ranstam Ph.D.[3], Ewa Roos P.T., Ph.D.[4], Per Aspenberg M.D.[2]*, Ph.D.[2] and Jörg Schilcher M.D., Ph.D.[2]

[1] Department of Orthopaedic Surgery, Capio Specialistvård Motala, Motala, Sweden

[2] Department of Orthopaedics and Department of Biomedical and Clinical Sciences, Faculty of Health Science, Linköping University, Linköping, Sweden

[3] Department of Clinical Sciences Lund, Orthopaedics, Lund University, Lund, Sweden

[4] Research Unit for Musculoskeletal Function and Physiotherapy, Institute of Sports Science and Clinical Biomechanics, University of Southern Denmark, Odense, Denmark

* Author deceased

Correspondence to
Dr Jörg Schilcher, Study Sponsor. Department of Orthopaedic Surgery, Linköping University Hospital, 58185 Linköping, Sweden, +46 10-103 43 12, jorg.schilcher@liu.se

Key words
Total joint replacement, Aseptic loosening, Patient reported outcome measures, Zoledronate

Word count
3276

ABSTRACT

Introduction

In Sweden roughly 3000 patients are reoperated each year due to pain and loss of function related to a loosened hip or knee prosthesis. These reoperations are strenuous for the patient, technically demanding and costly for the healthcare system. Any such reoperation that can be prevented would be of great benefit. Bisphosphonates are drugs that inhibit osteoclast function. Several clinical trials suggest that bisphosphonates lead to improved implant fixation and one small study even indicates better functional outcome. Furthermore, in epidemiological studies bisphosphonates have been shown to decrease the rate of revision for aseptic loosening by half. Thus, there are several indirect indications that bisphosphonates could improve patient reported outcome, but no firm evidence.

Methods and analysis

This is a pragmatic randomized, placebo-controlled, double-blinded, academic clinical trial of a single postoperative dose of zoledronic acid, in patients younger than 80 years undergoing primary total hip- or knee replacement for osteoarthritis. Participants will be recruited from two orthopaedic departments. All surgeries will be performed, and study drugs given at Motala Hospital. Sweden. The primary endpoint is to investigate between-group differences in the Hip dysfunction and Osteoarthritis Outcome Score (HOOS) and the Knee injury and Osteoarthritis Outcome Score (KOOS) at three years follow-up. Secondary outcomes will be investigated at one, three and six years, and stratified for hip and knee implants. These secondary endpoints are supportive, exploratory or explanatory. A total of 1000 patients will be included in the study.

Ethics and dissemination

The study has been approved by the Regional Ethical Review Board in Linköping (DNR 2015/286-31). The study will be reported in accordance with the Consolidated Standards of Reporting Trials statement for pharmacological trials. The results will be submitted for publication in peer-reviewed academic journals and disseminated to patient organisations and the media.

Registration details

The study is registered at EudraCT (No 2015-001200-55).

ARTICLE SUMMARY

Strengths and limitations of this study

- This is the first study to examine if a single intravenous dose zoledronic acid can improve patient reported outcome after primary total hip- or knee replacement.
- With 1000 patients included, this is the largest drug trial ever performed to test the effect of bisphosphonate treatment on the outcome after total joint replacement.
- The primary outcome variables HOOS and KOOS are well validated and aim to directly evaluate patient reported outcomes without the use of surrogate variables.
- Plain radiographs allow indirect assessment of treatment efficacy through radiographic evaluation of implant fixation as a secondary outcome.
- All patients are recruited from only two centres and operated at one single hospital, which might limit generalizability of the results.

For peer review only

INTRODUCTION

Total hip arthroplasty (THA) is one of the most successful operations ever invented and has been called “The operation of the century”.(1) Total knee arthroplasty (TKA) has similar success rates.(2, 3) When performed in elderly patients one can expect a less than 10% chance of ever needing secondary surgery.(4, 5) However, in Sweden roughly 3000 patients are operated on annually mainly because of pain and loss of function related to a loosened hip- or knee prosthesis.(4) These reoperations are often difficult for the surgeon and the patient, and the economic cost is several folds higher than for primary operation.(6) Also, results are less beneficial(7) and the complication rate is higher.(8) Any such reoperation that can be prevented would be of great benefit.

Implants loosen due to resorption of their bone bed by osteoclasts. When an implant is inserted into bone, a fracture healing response is activated.(9) This includes an increase in local bone formation and resorption, which are not necessarily coupled. If resorption outweighs bone formation the initial fixation of the implant might be impaired. This excessive motion between the implant and its surrounding bone bed (implant migration) might allow pressurized fluid flows and invasion of wear debris particles leading to further bone resorption.(10) When direct bone contact does not occur in the early postoperative period a fibrous tissue membrane will be formed leading to early subclinical loosening.(11, 12) The primary postoperative result, i.e. the fixation, can be estimated by specific radiographic methods (radiostereometry) to measure implant migration. There is a strong correlation between postoperative migration measured with radiostereometry and late loosening, showing that the early fixation is important for the late results. For acetabular cups the area under the receiver operating characteristic curve (ROC) for increased migration 2 years postoperatively to predict loosening after 10 years is 0.88 (95% confidence interval, 0.74 to 1.00).(13) Similar associations can be found for tibial components in total knee replacement.(14) This suggests that late loosening is the final result of a continuous process that starts immediately after the operation. Radiostereometry is partly invasive and very costly and can only be used in small series of patients.

Bisphosphonates specifically inhibit osteoclast activity, while in the fracture healing context bone formation remains increased. Therefore, bisphosphonate treatment at the time of implant insertion would possibly create a positive balance between bone formation and resorption leading to a net anabolic effect in the bone surrounding the implant thus leading to a more stable primary fixation.(15) Several randomised trials have shown that bisphosphonate treatment at the time of surgery reduces implant migration in TKA,(16) THA(17, 18) and dental implants.(19) However, the effect of zoledronic acid on uncemented femoral stems remains unclear.(20) One clinical trial comprising a small sample of younger patients (n=50) also reported an improved functional outcome on the Harris Hip Score.(17) All other RCT´s showed no effect of bisphosphonate treatment on patient reported outcome, but none of these trials were powered to detect such a difference. A recent meta-analysis of four epidemiological studies using hip and knee arthroplasty registries(21-24) has shown that bisphosphonate use is associated with a 50% decrease in the need for revision surgery.(25) Despite these findings, bisphosphonate treatment is not established in routine post-operative care to improve outcome after total joint replacement (TJR).

We here describe the study protocol for a pivotal trial designed to provide final evidence for the use of intravenous bisphosphonate to improve patient reported outcome after primary THA and TKA.

For peer review only - http://bmjopen.bmj.com/site/about/guidelines.xhtml

METHOD AND ANALYSIS

Study design

This is a single centre, pragmatic, randomized, placebo-controlled, double-blinded, academic clinical trial. Participants will be recruited from two orthopaedic departments in Region Östergötland, Sweden. The main centre for recruitment is Motala Hospital (Capio Specialistvård Motala from 1 April 2019, and previously Aleris Specialistvård Motala) where roughly 85% of all patients will be recruited. The remaining 15% will be recruited from the Department of Orthopaedic Surgery at Linköping University Hospital. All patients will be referred to both orthopaedic departments based on standard health care routines in Region Östergötland. All surgeries will be performed, and study drugs given at Motala Hospital, Sweden. Patients scheduled for primary hip- or knee arthroplasty, with respect given to inclusion and exclusion criteria, will be asked to participate both at the primary outpatient visit and after phone contact with a study nurse some weeks before the scheduled surgery. The final written consent will be given on the day of surgery (supplementary file 1). All other treatment outside the study protocol described here will be according to the clinical routines of the hospital. Inclusion of patients was started January 4th, 2016. Data collection for the primary outcome will continue until patients have been followed for three years, roughly until 2024.

Patients

1000 patients, 500 in each group, fulfilling the eligibility criteria (Table 1) will be included.

Table 1. Overview of inclusion and exclusion criteria

**Inclusion criteria**	All patients eligible for primary hip or knee prosthesis for any form of osteoarthritis, between 18 to 80 years of age.
**Exclusion criteria**	Previous or present use of bisphosphonates or other antiresorptives.
	Present use of other drugs which influence bone, e.g. anti-osteoporotic agents, glucocorticoids, anti-epileptics, or use less than a year before randomization.
	Present use of nephrotoxic medication.
	Active malignant disease.
	Pregnancy and breast feeding.
	Metabolic disease (other than osteoporosis) affecting the skeleton.
	Rheumatic disease.
	Hypocalcaemia as defined by local lab criteria.
	Simultaneous bilateral surgery.
	Communication problems (drug abuse, language or behaviour problems).
	Creatinine clearance (GFR) <35 mL/min.
	Regular use of corticosteroids more than 5 mg prednisolone per day.
	Atypical fracture or osteonecrosis of the jaw.
	Expected follow-op period less than 3 years (e.g. due to uncontrolled malignancy).
	Expected to require special postoperative surveillance due to increased surgical risk (e.g. for cardiac, psychiatric condition).

For peer review only

Randomization procedure and blinding

When found eligible, patients will be randomized to either zoledronic acid or placebo through block randomization by the study nurse on the day of surgery. Block randomization will be used to label infusion bags for drug delivery. The type of implant (hip or knee, cemented or not) will be a stratification factor in the randomization to ensure balance among these factors. All staff involved in patient care are blinded to treatment. The nurse in the postoperative care unit who is responsible for the preparation of the study drug according to the randomization list will not be blinded. However, because this person is not otherwise involved in the study, concealment of treatment allocation is not jeopardized. The content of the infusion bag will be administered to the patient on the day after surgery by a blinded nurse in the surgical ward. The randomization list will be available for unblinding in emergency situations 24 h a day at *Apoteksbolaget AB* at Linköping University Hospital.

Intervention

Patients will be randomized to receive a single postoperative infusion of zoledronic acid 4 mg/5 ml(17) or placebo (5ml saline) on the day after surgery.

Study Outcomes

Rationale for the outcome measures

In previous epidemiological studies of prosthetic loosening the endpoint has been revision surgery. We will report this parameter continuously and we will use the Swedish hip and knee arthroplasty registries to capture reoperations performed outside our uptake area. Since the overall revision rate for aseptic loosening in Sweden is around 2-3% during a 10-year period, this endpoint would demand not only a very large study sample but also a long-term follow-up to get sufficient power. Also, some patients with loosening do not undergo revision surgery. They might be too old or fragile for these demanding operations. Other patients only have modest symptoms and might refrain from a demanding reoperation. Therefore, another primary outcome must be considered for reasons of feasibility. A previous study with the same treatment protocol of zoledronic acid as ours reported not only less implant migration in the zoledronic acid group but also a statistically significant improvement on the Harris Hip Score(17) two and three years postoperatively, despite small numbers (n=50). This study comprised only uncemented prostheses in younger patients with osteonecrosis of the femoral head. Based on these findings, because of the clinical importance and the predictive value on future revision surgery, *(26-29)* we have chosen to use patient reported scores as our primary outcome: Hip dysfunction and Osteoarthritis Outcome Score (HOOS), Swedish version LK 2.0(30) and the Knee Injury and Osteoarthritis Outcome Score (KOOS), Swedish version LK1.0.(31) Both instruments were meticulously designed with items generated in an iterative process including input from stakeholder groups comprised of patients, orthopaedists, and physical therapists. Both instruments have undergone extensive psychometric testing(32) and are recommended for evaluation of TJR by the International Consortium for Health Outcomes Measurement. These measures are free to use and have previously been shown to be highly reliable, with excellent internal consistency (Cronbach's alpha coefficient of 0.82-0.98) in samples of people undergoing THR and TKR.(33, 34)

Our primary outcome measure will be between-group differences in KOOS/HOOS from baseline until the 3-year follow-up. Based on our literature review at the time of study design and confirmed later by the *Outcomes Measures in Rheumatology*, *Workgroup Total Joint Replacement*,(35) the subscale *pain* in HOOS/KOOS will be analysed as the primary endpoint in the confirmative analysis.

For peer review only

Secondary endpoints are included for supportive evidence. As secondary endpoints, we will analyse between-group differences in the remaining subscales of the KOOS/HOOS from baseline to 3 years, all subscales of the KOOS/HOOS and the RAND/SF36 (Swedish version from 2013-05-21, using the 4 week recall period)(36, 37) at 1, 3 and 6 years and signs of radiographic loosening at 3 and 6 years (Table 2). The RAND/SF36 will be analysed using physical and mental component scores.

For peer review only

Table 2. Schedule of assessments and events.

**Visit**	1	2	3	4	5	6	7	8
	Screening	Surgery	Treatment	Discharge	Follow-up	Follow-up	Follow-up	Follow-up
**Time**	Day -28 to - 2	Day 1	Day 2	Day 3-5	6 weeks	1 year ±1 month	3 year ±1 month	6 year ±1 month
**Assessment /event**								
Informed Consent*[1]	X							
Demography Medical history	X							
Physical Examination	X							
Height and Weight	X							
Assessment of inclusion and exclusion criteria	X	X						
Blood sampling	X		X					
Start of continuous daily Calcium*[2]			X					
Surgery		X						
Randomization*[3]		X	X					
Administration of IMP/Placebo*[4]			X					
HOOS/KOOS RAND/SF-36 questionnaire	X					X	X	X
X-ray		X					X	X
Concomitant Medication	X	X						
Routine follow-up (via phone)						X		
AE Assessment*[5]			X	X	X	X	X	X

IMP = investigational medical product, HOOS = Hip dysfunction and Osteoarthritis Outcome Score, KOOS = Knee injury and Osteoarthritis Outcome Score, SF-36 = 36 item Short Form, X-ray = Plain radiography, AE = Adverse Event, SAE = Severe Adverse Event.

*[1] The Informed Consent Form must be signed before any study related procedure

*[2] Vitamin D and calcium will be given daily in standard dosage from day 2 for 1 month.

*[3] Randomization has to be performed as close as possible prior to the first IMP infusion

*[4] Administration of IMP/Placebo will be given the day after surgery.

*[5] AE/SAE will be collected via a questionnaire at visit 5 and personal interview at 4. At visit 6, 7 and 8 only SAE will be collected via a questionnaire. Reminders will be given by phone.

For peer review only

For peer review only - http://bmjopen.bmj.com/site/about/guidelines.xhtml

Statistical analysis

Power

Both HOOS and KOOS ranges from 0 to 100. The minimal important change is often reported to be 8-10 points but this estimate is dependent on contextual factors such as patient age, intervention and time to follow-up, and according to the developers of KOOS (www.koos.nu) no generic value of the minimal important change is available for KOOS, or any other patient reported outcome measure. In a large sample a small statistically significant difference is still indicative of an important treatment effect, even if the difference is smaller than the minimal clinically important difference. At the time of the study design, average reported KOOS values for 3 years after TKR were not available, and sample size calculations therefore were based on average values 2 years after TKR: 84, (SD 14). Lacking official consensus on a recommended clinically relevant difference the research team decided upon a 3-point difference on the HOOS/KOOS scale after 3 years. These values would with Student's t-test and a two-sided significance level of 5 % yield 90 % power when 450 patients are included in each arm. To compensate for a 10% withdrawal, a further 50 patients would be needed, leading to a total of 1000 patients to include in a superiority trial.

Statistical analysis plan

Statistical analysis of the primary endpoint will be carried out using a mixed model repeated measurements ANOVA of the changes in KOOS/HOOS from baseline until 3-years follow-up with covariate adjustment for baseline values of HOOS/KOOS, and with implant type (hip, knee and cemented, uncemented) and age (continuous) as further covariates. As supportive endpoints, we will analyse HOOS/KOOS subscales at 6-years follow-up, signs of radiographic loosening at 3 and 6 years and SF/RAND36 at 1, 3 and 6 years. we will also perform subgroup analysis of men and women and implant types.

To reduce the risk of bias during interpretation, blinded results from the analyses (with study groups labelled as group A and group B) will be presented to all the authors, who will agree in writing on two alternative interpretations.(38) Thereafter, the data manager will break the randomization code.

As part of an exploratory analysis and to be able to define the clinical impact of our results we will perform a responder analysis comparing the proportion of patients who achieve a substantial clinical improvement on the subscale *pain* in HOOS/KOOS between the treatment and the control group.(39) HOOS and KOOS values depend on age, BMI and sex, which will be considered as cofactors in the analysis.

No interim analysis will be performed.

Safety

Concomitant drug treatment

After the infusion, oral supplements of vitamin D and calcium will be given once daily to both groups for the first postoperative month to prevent bisphosphonate-induced hypocalcaemia.

Zoledronic acid

Repeated infusions of zoledronic acid has been associated with a slight increase in atrial fibrillation in the highest age groups(40) and because of this we have set an upper age limit for inclusion to 80 years. Bisphosphonate use is strongly associated with osteonecrosis of the jaw. This is however a very rare condition and only associated with multiple dosing over

For peer review only

time.(41) 10% of patients treated with zoledronic acid have reported Acute-Phase Reactions (42) which can lead to a prolonged hospital stay. Even though zoledronic acid has not been reported to cause hypocalcaemia in osteoporosis treatment,(43) normal preoperative calcium and vitamin D levels are required for inclusion for safety reasons and patients will get oral supplements for the first postoperative month. Zoledronic acid can affect kidney function and the manufacturer recommends against its use in patients with creatinine clearance <35 ml/min.(44) Bisphosphonates are used in large scale to treat patients with osteoporosis, and its safety is extensively documented.(41) Furthermore, in this study only one dose of 4mg(17) is given, compared to the repeated dosing of 5mg in osteoporosis treatment.

Adverse events

Patients will be followed-up for 6 weeks after the infusion for adverse events (AE). The physiotherapist will record AE´s at the 6-week follow-up (Table 2). An AE is defined in the International Conference on Harmonization (ICH) Guidelines for Good Clinical Practice as "any untoward medial occurrence in a patient or clinical investigation subject administered a pharmaceutical product and does not necessarily have a causal relationship with this treatment". The occurrence of atypical femoral fractures and osteonecrosis of the jaw will be recorded throughout the whole study period. Serious adverse event (SAE) is defined as an AE that is fatal, life threatening, requires in-patient hospitalization or prolonged hospitalization, results in persistent or significant disability/incapacity or other significant medical hazards. Adverse Drug Reaction (ADR) is defined as all untoward and unintended response to a medical product related to any dose administered and will also be recorded during the first 6 weeks. The occurrence of AE and SAE will be presented descriptively. For any harm caused through study participation, all patients are covered by the national Swedish patient insurance, *Landstingens Ömsesidiga Försäkringsbolag.*

ETHICS AND DISSEMINATION

Ethics

The study will be conducted in agreement with the Helsinki declaration and ICH guidelines will be adhered to. The study will be monitored by *Forum Östergötland,* which is part of the national organisation for Clinical Studies in Sweden, Forum Sydost. All completed questionnaires will be kept secured from unauthorized access within the research nurses´ facility. Data for the purpose of statistical analyses will be collected in digitized files. Other data will be stored in the patients´ ordinary medical chart. The study has been approved by the Regional Ethical Review Board in Linköping (DNR 2015/286-31).

Dissemination

The results of this study will be submitted for publication in an international peer reviewed scientific paper regardless of whether the results are positive, negative or inconclusive regarding the hypothesis of the study.

Patient and Public Involvement

No patient organisation or patient representatives were involved in the design of the study. The results of our study will be disseminated to patient organizations and the public through the Swedish Orthopaedic Association and the Swedish National Joint Arthroplasty Register.

For peer review only

DISCUSSION

Strengths and limitations

Strengths

The main strength of this study is its size and design. To our knowledge this is the largest RCT designed to elucidate if bisphosphonates can improve outcome in primary THA and TKA. If we can demonstrate a significant increase in patient satisfaction after bisphosphonate administration, it could revolutionize the perioperative care of patients undergoing TJR.

Limitations

The main limitation of this study can be considered its primary patient-reported outcome measure. A hard endpoint as a prospectively collected rate of revision would be preferable, however not feasible in this research question. Also, not all patients with loosening of their implants undergo revision surgery. Some are too old and fragile, and others have moderate symptoms and might decide to abstain from surgery. The use of a patient-reported primary endpoint will increase the relevance of the findings to patients. Improved patient reported outcomes after TJR might in fact be more important for the majority of the patients undergoing TJR compared to prosthetic loosening assessed on radiographs, which is a secondary outcome. If we fail to demonstrate a significant increase in patient reported outcomes, we might be able to show a decrease in radiographic signs of early loosening, which strongly correlates with late aseptic loosening.(13,14) Also, dual-energy x-ray absorptiometry will not be performed.

Authors affiliation

1 Department of Orthopaedic Surgery, Capio Specialistvård Motala, Motala, Sweden

2 Department of Orthopaedics and Experimental and Clinical Medicine, Faculty of Health Science, Linköping University, Linköping, Sweden

3 Department of Clinical Sciences Lund, Orthopaedics, Lund University, Lund, Sweden

4 Research Unit for Musculoskeletal Function and Physiotherapy, Institute of Sports Science and Clinical Biomechanics, University of Southern Denmark, Odense, Denmark

Author contributions

JB: Writing of manuscript, study design
HL: Writing of manuscript, study design
JR: Revision of manuscript, study design
ER: Revision of manuscript, study design
PA: Study design, preparation of manuscript
JS: Writing and revision of manuscript, study design

Funding statement

This academic drug trial is run without any support from the industry. Financial support was received from the Swedish Research Council, grant number 2014-07284, and ALF-grants from Region Östergötland, Sweden.

For peer review only

Data statement

The dataset will be made available in a data repository.

Competing interests

None stated. Data collection, management, analysis, and interpretation of data; writing of the report; and the decision to submit the report for publication are the sole authority of the study sponsor.

Acknowledgements

We are grateful for the research-friendly leadership of the healthcare providers Capio Specialistvård Motala and Aleris Specialistvård Motala. We also thank all personnel at Motala hospital for their extra effort to make this large research project possible, in specific our research nurses Anneli Kvarnestedt and Carmen Henriksson.

REFERENCES

1. Learmonth ID, Young C, Rorabeck C. The operation of the century: total hip replacement. *Lancet* 2007;370:1508-19.
2. Evans JT, Walker RW, Evans JP, et al. How long does a knee replacement last? A systematic review and meta-analysis of case series and national registry reports with more than 15 years of follow-up. *The Lancet* 2019;393:655-63.
3. Beswick AD, Wylde V, Gooberman-Hill R, et al. What proportion of patients report long-term pain after total hip or knee replacement for osteoarthritis? A systematic review of prospective studies in unselected patients. *BMJ Open* 2012;2:e000435.
4. Kärrholm J MM, Odin M, Vinblad J, Rogmark C, Rolfson O. The Swedish Hip Arthroplasty Register, Annual Report 2017. 2018.
5. Robertsson O LL, Sundberg M, W-Dahl A,. The Swedish Knee Arthroplasty Register, Annual Report 2019. 2019.
6. Vanhegan IS, Malik AK, Jayakumar P, et al. A financial analysis of revision hip arthroplasty: the economic burden in relation to the national tariff. *The Journal of bone and joint surgery British volume* 2012;94:619-23.
7. Lenguerrand E, Whitehouse MR, Wylde V, et al. Pain and Function Recovery Trajectories following Revision Hip Arthroplasty: Short-Term Changes and Comparison with Primary Hip Arthroplasty in the ADAPT Cohort Study. *PloS one* 2016;11:e0164839.
8. Ong KL, Lau E, Suggs J, et al. Risk of subsequent revision after primary and revision total joint arthroplasty. *Clin Orthop Relat Res* 2010;468:3070-6.
9. Bernhardsson M, Sandberg O, Aspenberg P. Experimental models for cancellous bone healing in the rat. *Acta Orthop* 2015;86:745-50.
10. Aspenberg P, Van der Vis H. Migration, particles, and fluid pressure. A discussion of causes of prosthetic loosening. *Clinical orthopaedics and related research* 1998:75-80.
11. Schindeler A, McDonald MM, Bokko P, et al. Bone remodeling during fracture repair: The cellular picture. *Semin Cell Dev Biol* 2008;19:459-66.
12. Agholme F, Aspenberg P. Experimental results of combining bisphosphonates with allograft in a rat model. *J Bone Joint Surg Br* 2009;91:670-5.
13. Pijls BG, Nieuwenhuijse MJ, Fiocco M, et al. Early proximal migration of cups is associated with late revision in THA: a systematic review and meta-analysis of 26 RSA studies and 49 survivalstudies. *Acta Orthop* 2012;83:583-91.
14. Pijls BG, Valstar ER, Nouta KA, et al. Early migration of tibial components is associated with late revision: a systematic review and meta-analysis of 21,000 knee arthroplasties. *Acta Orthop* 2012;83:614-24.
15. Yu NY, Schindeler A, Tagil M, et al. Use of BMPs and bisphosphonates in improving bone fracture healing. *Front Biosci (Elite Ed)* 2012;4:2647-53.

For peer review only

16. Hilding M, Aspenberg P. Local peroperative treatment with a bisphosphonate improves the fixation of total knee prostheses: a randomized, double-blind radiostereometric study of 50 patients. *Acta Orthop* 2007;78:795-9.
17. Friedl G, Radl R, Stihsen C, et al. The effect of a single infusion of zoledronic acid on early implant migration in total hip arthroplasty. A randomized, double-blind, controlled trial. *The Journal of bone and joint surgery American volume* 2009;91:274-81.
18. Schilcher J, Palm L, Ivarsson I, et al. Local bisphosphonate reduces migration and formation of radiolucent lines adjacent to cemented acetabular components. *Bone Joint J* 2017;99-b:317-24.
19. Abtahi J, Tengvall P, Aspenberg P. A bisphosphonate-coating improves the fixation of metal implants in human bone. A randomized trial of dental implants. *Bone* 2012;50:1148-51.
20. Aro E, Moritz N, Mattila K, et al. A long-lasting bisphosphonate partially protects periprosthetic bone, but does not enhance initial stability of uncemented femoral stems: A randomized placebo-controlled trial of women undergoing total hip arthroplasty. *J Biomech* 2018;75:35-45.
21. Thillemann TM, Pedersen AB, Mehnert F, et al. Postoperative use of bisphosphonates and risk of revision after primary total hip arthroplasty: a nationwide population-based study. *Bone* 2010;46:946-51.
22. Prieto-Alhambra D, Javaid MK, Judge A, et al. Association between bisphosphonate use and implant survival after primary total arthroplasty of the knee or hip: population based retrospective cohort study. *BMJ* 2011;343:d7222.
23. Prieto-Alhambra D, Lalmohamed A, Abrahamsen B, et al. Oral bisphosphonate use and total knee/hip implant survival: validation of results in an external population-based cohort. *Arthritis & rheumatology* 2014;66:3233-40.
24. Khatod M, Inacio MC, Dell RM, et al. Association of Bisphosphonate Use and Risk of Revision After THA: Outcomes From a US Total Joint Replacement Registry. *Clin Orthop Relat Res* 2015;473:3412-20.
25. Teng S, Yi C, Krettek C, et al. Bisphosphonate Use and Risk of Implant Revision after Total Hip/Knee Arthroplasty: A Meta-Analysis of Observational Studies. *PloS one* 2015;10:e0139927.
26. Rothwell AG, Hooper GJ, Hobbs A, et al. An analysis of the Oxford hip and knee scores and their relationship to early joint revision in the New Zealand Joint Registry. *The Journal of bone and joint surgery British volume* 2010;92:413-8.
27. Rolfson O, Bohm E, Franklin P, et al. Patient-reported outcome measures in arthroplasty registries Report of the Patient-Reported Outcome Measures Working Group of the International Society of Arthroplasty Registries Part II. Recommendations for selection, administration, and analysis. *Acta Orthop* 2016;87 Suppl 1:9-23.
28. Devane P, Horne G, Gehling DJ. Oxford hip scores at 6 months and 5 years are associated with total hip revision within the subsequent 2 years. *Clinical orthopaedics and related research* 2013;471:3870-4.
29. Singh JA, Schleck C, Harmsen S, et al. Clinically important improvement thresholds for Harris Hip Score and its ability to predict revision risk after primary total hip arthroplasty. *BMC Musculoskelet Disord* 2016;17:256.
30. Nilsdotter AK, Lohmander LS, Klassbo M, et al. Hip disability and osteoarthritis outcome score (HOOS)--validity and responsiveness in total hip replacement. *BMC Musculoskelet Disord* 2003;4:10.
31. Roos EM, Toksvig-Larsen S. Knee injury and Osteoarthritis Outcome Score (KOOS) - validation and comparison to the WOMAC in total knee replacement. *Health Qual Life Outcomes* 2003;1:17.
32. Losina E, Ranstam J, Collins JE, et al. OARSI Clinical Trials Recommendations: Key analytic considerations in design, analysis, and reporting of randomized controlled trials in osteoarthritis. *Osteoarthritis and cartilage* 2015;23:677-85.
33. Nilsdotter A, Bremander A. Measures of hip function and symptoms: Harris Hip Score (HHS), Hip Disability and Osteoarthritis Outcome Score (HOOS), Oxford Hip Score (OHS), Lequesne Index of Severity for Osteoarthritis of the Hip (LISOH), and American Academy of Orthopedic Surgeons (AAOS) Hip and Knee Questionnaire. *Arthritis Care Res (Hoboken)* 2011;63 Suppl 11:S200-7.

For peer review only

34. Collins NJ, Roos EM. Patient-reported outcomes for total hip and knee arthroplasty: commonly used instruments and attributes of a "good" measure. *Clinics in geriatric medicine* 2012;28:367-94.
35. Singh JA, Dowsey MM, Dohm M, et al. Achieving Consensus on Total Joint Replacement Trial Outcome Reporting Using the OMERACT Filter: Endorsement of the Final Core Domain Set for Total Hip and Total Knee Replacement Trials for Endstage Arthritis. *The Journal of rheumatology* 2017;44:1723-6.
36. Hays RD, Sherbourne CD, Mazel RM. The RAND 36-Item Health Survey 1.0. *Health Econ* 1993;2:217-27.
37. Ware JE, Jr., Gandek B. Overview of the SF-36 Health Survey and the International Quality of Life Assessment (IQOLA) Project. *J Clin Epidemiol* 1998;51:903-12.
38. Jarvinen TL, Sihvonen R, Bhandari M, et al. Blinded interpretation of study results can feasibly and effectively diminish interpretation bias. *J Clin Epidemiol* 2014;67:769-72.
39. Lyman S, Lee YY, McLawhorn AS, et al. What Are the Minimal and Substantial Improvements in the HOOS and KOOS and JR Versions After Total Joint Replacement? *Clinical orthopaedics and related research* 2018;476:2432-41.
40. Kim DH, Rogers JR, Fulchino LA, et al. Bisphosphonates and risk of cardiovascular events: a meta-analysis. *PLoS One* 2015;10:e0122646.
41. Reyes C, Hitz M, Prieto-Alhambra D, et al. Risks and Benefits of Bisphosphonate Therapies. *J Cell Biochem* 2016;117:20-8.
42. Kotian P, Boloor A, Sreenivasan S. Study of Adverse Effect Profile of Parenteral Zoledronic Acid in Female Patients with Osteoporosis. *J Clin Diagn Res* 2016;10:OC04-6.
43. Kreutle V, Blum C, Meier C, et al. Bisphosphonate induced hypocalcaemia - report of six cases and review of the literature. *Swiss Med Wkly* 2014;144:w13979.
44. McClung M, Harris ST, Miller PD, et al. Bisphosphonate therapy for osteoporosis: benefits, risks, and drug holiday. *Am J Med* 2013;126:13-20.

For peer review only

Patientens namn: ………………………

Studienummer: ………………………

Patientinformation

Du har artros i en höft- eller knäled. Tillsammans med din behandlande läkare har ni kommit fram till att du behöver opereras med en protes.
Denna patientinformation är en förfrågan om att delta i en forskningsstudie.

Zoledronat är ett läkemedel registrerat för behandling av benskörhet. Det verkar genom att minska den naturliga nedbrytningen av skelettet som sker med åldern. Läkemedlet verkar också kunna förbättra fastläkning av ledproteser.

Syftet med denna studie är att ta reda på om Zoledronat kan förbättra resultat efter protesoperationer i höft- eller knäled. Vi planerar att inkludera 1000 patienter som behöver en höft- eller knäprotes på grund artros. Hälften av patienterna får behandling med Zoledronat dagen efter operation och den andra hälften får endast koksalt (overksamt läkemedel). Behandlingen sker genom en långsam injektion (infusion). I övrigt påverkas inte ditt omhändertagande före eller efter operationen, utöver att vi kommer att kontrollera den opererade leden med röntgen efter 3 år och 6 år. Du kommer också att få 2 formulär hemskickat till dig vid 1, 3 och 6 år efter operation. Med dessa formulär vill vi på ett standardiserat sätt få reda på hur du upplever resultatet av din operation.

Behandling med Zoledronat kan ge biverkningar. Vanliga biverkningar efter den första infusionen är feber och huvudvärk (influensaliknande). De flesta av dessa biverkningar uppträder inom de tre första dagarna efter behandlingen och upphör inom 3 dagar efter att de började. Genom den smärtlindring du får på grund av operationen kommer du sannolikt inte märka dessa biverkningar alls. Sällsynta och allvarliga biverkningar vid behandling med Zoledronat är frakturer i lårbenet och nedbrytning av käkbenet. Dessa förekommer dock endast vid upprepade behandlingar och i kombination med allvarliga grundsjukdomar. I denna studie ges zoledronat som en engångsdos, dagen efter din operation.

Om du vill delta, kommer du att lottas till antingen behandling med placebo (koksalt) eller Zoledronat. Varken Du eller din opererande läkare vet vilken behandling Du får.

Ditt deltagande är helt frivilligt. Om du väljer att inte delta kommer du att omhändertas på sedvanligt sätt. Du kan när som helst under studien avbryta ditt deltagande utan att det påverkar din behandling.

Version 1.5 2019-04-01

For peer review only

Om Du beslutar dig för att avbryta studien, kommer din doktor att be dig om tillåtelse att samla in information från dina journalhandlingar. Studien är godkänd av Etikprövningsnämnd och tillstånd har lämnats av Läkemedelsverket. Du är som patient försäkrad genom Läkemedelsförsäkringen och Patientskadelagen.

Behandling av personuppgifter

Under studien kommer ansvarig läkare att samla in uppgifter om födelsedatum, kön, hälsodata (såsom t.ex. tidigare sjukdomar och läkemedelsanvändning) samt resultat av undersökningar i studien.

Uppgifter insamlas i studien utan ditt namn eller personnummer men med en kod. Endast ansvarig läkare har tillgång till din ”nyckel”, med vilken det går att koppla uppgifterna till dig. Capio Specialistvård i Motala AB är personuppgifts-ansvariga för behandling av personuppgifter.

Ändamålen med detta register är forskning och utveckling av läkemedel som beskrivits i denna information samt godkännande/registrering av kommande produkter och säkerhetsuppföljning, därmed är allmänt intresse den rättsliga grunden för hantering av personuppgifter. Resultat kan också komma att publiceras i någon medicinsk tidskrift utan att din identitet uppges.

Uppgifterna hanteras enligt Dataskyddsförordningen, GDPR (EU 2016/679) och du har rätt att få veta vilka uppgifter som samlas in om dig, begära rättelse vid eventuella felaktigheter eller begära begränsning/ borttagning av uppgifter.

Ansvarig läkare:
Håkan Ledin, Mobil: 072-204 56 35; hakan.ledin@regionostergotland.se
Bengt Horn, Mobil: 070-158 63 54 Bengt.Horn.af.Aminne@regionostergotland.se

Skriftligt Samtycke

Jag har tagit del av informationen och accepterar att deltaga i studien. Jag har också informerats om och samtyckt till att en oberoende granskare (monitor) och läkemedelsmyndighet vid behov får jämföra de i studien rapporterade uppgifterna med de som finns i min patientjournal. Detta får ske under förbehåll att den information som då blir tillgänglig inte förs vidare.

____

Underskrift Patient Namnförtydligande Datum

____

Underskrift Läkare Namnförtydligande Datum

Version 1.5 2019-04-01

For peer review only

Reporting checklist for protocol of a clinical trial.

Based on the SPIRIT guidelines.

Instructions to authors

Complete this checklist by entering the page numbers from your manuscript where readers will find each of the items listed below.

Your article may not currently address all the items on the checklist. Please modify your text to include the missing information. If you are certain that an item does not apply, please write "n/a" and provide a short explanation.

Upload your completed checklist as an extra file when you submit to a journal.

In your methods section, say that you used the SPIRITreporting guidelines, and cite them as:

Chan A-W, Tetzlaff JM, Altman DG, Laupacis A, Gøtzsche PC, Krleža-Jerić K, Hróbjartsson A, Mann H, Dickersin K, Berlin J, Doré C, Parulekar W, Summerskill W, Groves T, Schulz K, Sox H, Rockhold FW, Rennie D, Moher D. SPIRIT 2013 Statement: Defining standard protocol items for clinical trials. Ann Intern Med. 2013;158(3):200-207

		Reporting Item	Page Number
**Administrative information**			
Title	#1	Descriptive title identifying the study design, population, interventions, and, if applicable, trial acronym	1
Trial registration	#2a	Trial identifier and registry name. If not yet registered, name of intended registry	2
Trial registration: data set	#2b	All items from the World Health Organization Trial Registration Data Set	2
Protocol version	#3	Date and version identifier	2
Funding	#4	Sources and types of financial, material, and other support	10
Roles and responsibilities: contributorship	#5a	Names, affiliations, and roles of protocol contributors	9

For peer review only

Roles and responsibilities: sponsor contact information	#5b	Name and contact information for the trial sponsor	1
Roles and responsibilities: sponsor and funder	#5c	Role of study sponsor and funders, if any, in study design; collection, management, analysis, and interpretation of data; writing of the report; and the decision to submit the report for publication, including whether they will have ultimate authority over any of these activities	9
Roles and responsibilities: committees	#5d	Composition, roles, and responsibilities of the coordinating centre, steering committee, endpoint adjudication committee, data management team, and other individuals or groups overseeing the trial, if applicable (see Item 21a for data monitoring committee)<br><br>**No committees involved**	n/a
**Introduction**			
Background and rationale	#6a	Description of research question and justification for undertaking the trial, including summary of relevant studies (published and unpublished) examining benefits and harms for each intervention	3
Background and rationale: choice of comparators	#6b	Explanation for choice of comparators	5
Objectives	#7	Specific objectives or hypotheses	3
Trial design	#8	Description of trial design including type of trial (eg, parallel group, crossover, factorial, single group), allocation ratio, and framework (eg, superiority, equivalence, non-inferiority, exploratory)	3
**Methods: Participants, interventions, and outcomes**			
Study setting	#9	Description of study settings (eg, community clinic, academic hospital) and list of countries where data will be collected. Reference to where list of study sites can be obtained	3

For peer review only

Eligibility criteria	#10	Inclusion and exclusion criteria for participants. If applicable, eligibility criteria for study centres and individuals who will perform the interventions (eg, surgeons, psychotherapists)	4
Interventions: description	#11a	Interventions for each group with sufficient detail to allow replication, including how and when they will be administered	4
Interventions: modifications	#11b	Criteria for discontinuing or modifying allocated interventions for a given trial participant (eg, drug dose change in response to harms, participant request, or improving / worsening disease)<br>**Only one dose is given**	n/a
Interventions: adherance	#11c	Strategies to improve adherence to intervention protocols, and any procedures for monitoring adherence (eg, drug tablet return; laboratory tests)	6
Interventions: concomitant care	#11d	Relevant concomitant care and interventions that are permitted or prohibited during the trial	4
Outcomes	#12	Primary, secondary, and other outcomes, including the specific measurement variable (eg, systolic blood pressure), analysis metric (eg, change from baseline, final value, time to event), method of aggregation (eg, median, proportion), and time point for each outcome. Explanation of the clinical relevance of chosen efficacy and harm outcomes is strongly recommended	5
Participant timeline	#13	Time schedule of enrolment, interventions (including any run-ins and washouts), assessments, and visits for participants. A schematic diagram is highly recommended (see Figure)	6
Sample size	#14	Estimated number of participants needed to achieve study objectives and how it was determined, including clinical and statistical assumptions supporting any sample size calculations	7
Recruitment	#15	Strategies for achieving adequate participant enrolment to reach target sample size	4
**Methods: Assignment of interventions (for controlled trials)**			
Allocation: sequence generation	#16a	Method of generating the allocation sequence (eg, computer-generated random numbers), and list of any factors for	4

For peer review only

		stratification. To reduce predictability of a random sequence, details of any planned restriction (eg, blocking) should be provided in a separate document that is unavailable to those who enrol participants or assign interventions	
Allocation concealment mechanism	#16b	Mechanism of implementing the allocation sequence (eg, central telephone; sequentially numbered, opaque, sealed envelopes), describing any steps to conceal the sequence until interventions are assigned	4
Allocation: implementation	#16c	Who will generate the allocation sequence, who will enrol participants, and who will assign participants to interventions	4
Blinding (masking)	#17a	Who will be blinded after assignment to interventions (eg, trial participants, care providers, outcome assessors, data analysts), and how	4
Blinding (masking): emergency unblinding	#17b	If blinded, circumstances under which unblinding is permissible, and procedure for revealing a participant's allocated intervention during the trial	4
**Methods: Data collection, management, and analysis**			
Data collection plan	#18a	Plans for assessment and collection of outcome, baseline, and other trial data, including any related processes to promote data quality (eg, duplicate measurements, training of assessors) and a description of study instruments (eg, questionnaires, laboratory tests) along with their reliability and validity, if known. Reference to where data collection forms can be found, if not in the protocol	6
Data collection plan: retention	#18b	Plans to promote participant retention and complete follow-up, including list of any outcome data to be collected for participants who discontinue or deviate from intervention protocols	6
Data management	#19	Plans for data entry, coding, security, and storage, including any related processes to promote data quality (eg, double data entry; range checks for data values). Reference to where details of data management procedures can be found, if not in the protocol	9

For peer review only

Statistics: outcomes	#20a	Statistical methods for analysing primary and secondary outcomes. Reference to where other details of the statistical analysis plan can be found, if not in the protocol	5
Statistics: additional analyses	#20b	Methods for any additional analyses (eg, subgroup and adjusted analyses)	5
Statistics: analysis population and missing data	#20c	Definition of analysis population relating to protocol non-adherence (eg, as randomised analysis), and any statistical methods to handle missing data (eg, multiple imputation)	5
**Methods: Monitoring**			
Data monitoring: formal committee	#21a	Composition of data monitoring committee (DMC); summary of its role and reporting structure; statement of whether it is independent from the sponsor and competing interests; and reference to where further details about its charter can be found, if not in the protocol. Alternatively, an explanation of why a DMC is not needed	8
Data monitoring: interim analysis	#21b	Description of any interim analyses and stopping guidelines, including who will have access to these interim results and make the final decision to terminate the trial	7
Harms	#22	Plans for collecting, assessing, reporting, and managing solicited and spontaneously reported adverse events and other unintended effects of trial interventions or trial conduct	8
Auditing	#23	Frequency and procedures for auditing trial conduct, if any, and whether the process will be independent from investigators and the sponsor	8
**Ethics and dissemination**			
Research ethics approval	#24	Plans for seeking research ethics committee / institutional review board (REC / IRB) approval	8
Protocol amendments	#25	Plans for communicating important protocol modifications (eg, changes to eligibility criteria, outcomes, analyses) to relevant parties (eg, investigators, REC / IRBs, trial participants, trial 8registries, journals, regulators)	8

For peer review only

Consent or assent	#26a	Who will obtain informed consent or assent from potential trial participants or authorised surrogates, and how (see Item 32)	3
Consent or assent: ancillary studies	#26b	Additional consent provisions for collection and use of participant data and biological specimens in ancillary studies, if applicable	
Confidentiality	#27	How personal information about potential and enrolled participants will be collected, shared, and maintained in order to protect confidentiality before, during, and after the trial	8
Declaration of interests	#28	Financial and other competing interests for principal investigators for the overall trial and each study site	9
Data access	#29	Statement of who will have access to the final trial dataset, and disclosure of contractual agreements that limit such access for investigators	9
Ancillary and post trial care	#30	Provisions, if any, for ancillary and post-trial care, and for compensation to those who suffer harm from trial participation	9
Dissemination policy: trial results	#31a	Plans for investigators and sponsor to communicate trial results to participants, healthcare professionals, the public, and other relevant groups (eg, via publication, reporting in results databases, or other data sharing arrangements), including any publication restrictions	9
Dissemination policy: authorship	#31b	Authorship eligibility guidelines and any intended use of professional writers	9
Dissemination policy: reproducible research	#31c	Plans, if any, for granting public access to the full protocol, participant-level dataset, and statistical code	9
**Appendices**			
Informed consent materials	#32	Model consent form and other related documentation given to participants and authorised surrogates	4
Biological specimens	#33	Plans for collection, laboratory evaluation, and storage of biological specimens for genetic or molecular analysis in the current trial and for future use in ancillary studies, if applicable<br>**No samples are stored for study purposes**	n/a

For peer review only

None The SPIRIT checklist is distributed under the terms of the Creative Commons Attribution License CC-BY-ND 3.0. This checklist can be completed online using https://www.goodreports.org/, a tool made by the EQUATOR Network in collaboration with Penelope.ai

For peer review only
